# Household perceptions and subjective valuations of indoor residual spraying programmes to control malaria in northern Uganda

**DOI:** 10.1186/s40249-016-0190-1

**Published:** 2016-10-06

**Authors:** Zachary S. Brown, Randall A. Kramer, David Ocan, Christine Oryema

**Affiliations:** 1 Department of Agricultural and Resource Economics, North Carolina State University, Raleigh, NC USA; 2 Nicholas School of the Environment, Duke University, Durham, NC USA; 3 Duke Global Health Institute, Duke University, Durham, NC USA; 4 Gulu University, Gulu Town, Uganda

## Abstract

**Background:**

Insecticide-based tools remain critical for controlling vector-borne diseases in Uganda. Securing public support from targeted populations for such tools is an important component in sustaining their long-run effectiveness. Yet little quantitative evidence is available on the perceived benefits and costs of vector control programmes among targeted households.

**Methods:**

A survey was administered to a clustered random sample of 612 households in Gulu and Oyam districts of northern Uganda during a period of very high malaria transmission and following a pilot indoor residual spray (IRS) programme. A discrete choice experiment was conducted within the survey, in which respondents indicated their preferences for different IRS programmes relative to money compensation in a series of experimentally controlled, hypothetical choice sets. The data were analysed using conditional logit regression models to estimate respondents’ willingness to accept (WTA) some amount of money compensation in lieu of foregone malaria risk reductions. Latent class models were used to analyse whether respondent characteristics predicted WTA.

**Results:**

Average WTA is estimated at $8.94 annually for a 10 % reduction in malaria risk, and additional co-benefits of IRS were estimated to be worth on average $54–$56 (depending on insecticide type) per round of IRS. Significant heterogeneity is observed: Four in five household heads in northern Uganda have high valuations for IRS programmes, while the remaining 20 % experience costly side effects of IRS (valued at between $2 and $3 per round). Statistically significant predictors of belonging to the high-value group include respondent gender, mean age of household members, participation in previous IRS, basic knowledge of mosquito reproduction, and the number of mosquito nets owned. Proxies for household income and wealth are not found to be statistically significant predictors of WTA.

**Conclusions:**

This study suggests that the majority of people in areas of high malaria transmission like northern Uganda place a high value on vector control programmes using IRS. However, there is significant heterogeneity in terms of the perceived side effects (positive and negative). This has implications for sustaining public support for these programmes in the long-term.

**Electronic supplementary material:**

The online version of this article (doi:10.1186/s40249-016-0190-1) contains supplementary material, which is available to authorized users.

## Multilingual abstracts

Please see Additional file 1 for translations of the abstract into the five official working language of the United Nations.

## Background

Insecticide-based suppression of malaria vectors has comprised a foundational pillar of malaria control programmes for over six decades in Uganda. Either applied to dwellings via indoor residual spraying (IRS) or used in insecticide-treated bed nets (ITNs), insecticides remain among the most cost-effective tools for reducing the health burden of malaria [1]. Recent evidence on the effectiveness of these tools suggests that, between 2000 and 2010, the prevalence of *Plasmodium falciparum* in Africa among populations between 2 and 10 years of age declined from approximately 33 % to 17 %, with ITNs and IRS accounting for roughly 60 % of this decrease [2].

While policymakers generally assume public support for malaria control programmes due to these manifest disease reduction benefits, there has been a persistent debate in academic and policy communities about the sustainability of continuing these *status quo* control methods indefinitely [3–5]. This question of sustainability often centres on the threat of insecticide resistance [6, 7], as well as the potential long-term health and environmental impacts from prolonged exposure to insecticides in the context of these vector control methods [8–10]. There is a growing evidence base analysing the biological dynamics involved in the spread of insecticide resistance [11, 12] and evaluating its operational impacts on vector control programmes [13–15]. Likewise, there is a significant toxicological literature measuring the potential health effects of household insecticide exposure in the context of malaria control [16–18].

Less research has quantitatively assessed these potential tradeoffs in terms of the preferences of the primary clients of vector control programmes, that is the targeted households themselves. Among this small literature are studies examining households’ perceptions of IRS programmes [19–21], as well as research evaluating households’ willingness to pay (WTP) for ITNs [22, 23]. However, we are aware of no prior studies which quantitatively evaluate households’ economic tradeoffs from participating in insecticide-based vector control, including the benefits of variable malaria risk reductions and the potential costs of household members’ exposure to insecticides, as well as other nonmonetary costs.

Examining households’ support and participation determinants is important, in particular, for managing insecticide resistance risks. A central principle in insect resistance management (IRM) has been the use of high-dose strategies using multiple insecticides [24, 25]. Household decisions are thus also an important component in successful IRM, because they mediate the effective dosage to which vector populations are exposed. Sustaining household support for insecticide-based vector control should therefore be a key ingredient in a successful IRM strategy in a vector control context.

We measure northern Ugandan households’ perceived money value of IRS programmes, by estimating their willingness to accept (WTA) money in lieu of different types of IRS programmes. An important advantage of this value measure over WTP in a developing country context is that, while it may be related to household income, it is not bounded by household budget constraints: impoverished households can still have a high WTA for vector control, even if their budget constraint does not permit a high WTP [26]. In public policy, WTP is often used in benefit-cost analysis (BCA) to measure values to those who stand to gain from a policy change, whereas WTA should in principle be used to value the damages to those experiencing a net loss as a result of the policy change [27]. Thus, our estimates of WTA in this context should be considered with caution if deployed in a BCA, being most appropriate for a situation in which a policy change would increase malaria risk levels (e.g. due to withdrawn government or donor funding for malaria control programmes). However, independent of their potential use in BCA analysis, our estimates do provide a unique economic perspective on impoverished households’ valuations of malaria risk reductions, without the confounding factor of a household budget constraint.

Prior to our study, one round of IRS had been conducted between February and April 2008 in Gulu and Oyam districts of northern Uganda. These programmes have been principally funded by the U.S. President’s Malaria Initiative (PMI) and are performed in coordination with the Ugandan National Malaria Control Program (NMCP). Gulu was sprayed using the pyrethroid-type insecticide lambda-cyhalothrin (which went by the trade-name ICON in the study region), and Oyam was sprayed using DDT [28]. IRS services were provided free-of-charge to households. However, there were potentially significant nonmonetary costs for households to participate, including an obligation to retrieve 10 l of water with which to mix the insecticide, the necessity of removing all household belongings and remaining outside of the home for at least two hours, as well as any perceived negative health effects from insecticides applied in the home.

We applied an econometric methodology to assess households’ valuations of these programmes. Using data from a survey-based economic experiment conducted among 612 randomly sampled households spread over two districts of northern Uganda, we evaluated monetary tradeoffs in terms of WTA between hypothetical IRS programmes that varied according to the type of chemical employed, the projected reduction in malaria risk obtained from the programme, and spray frequency. The data were collected as part of a broader survey of knowledge and attitudes regarding malaria vector control programmes [28].

## Methods

**Fig. 1:**
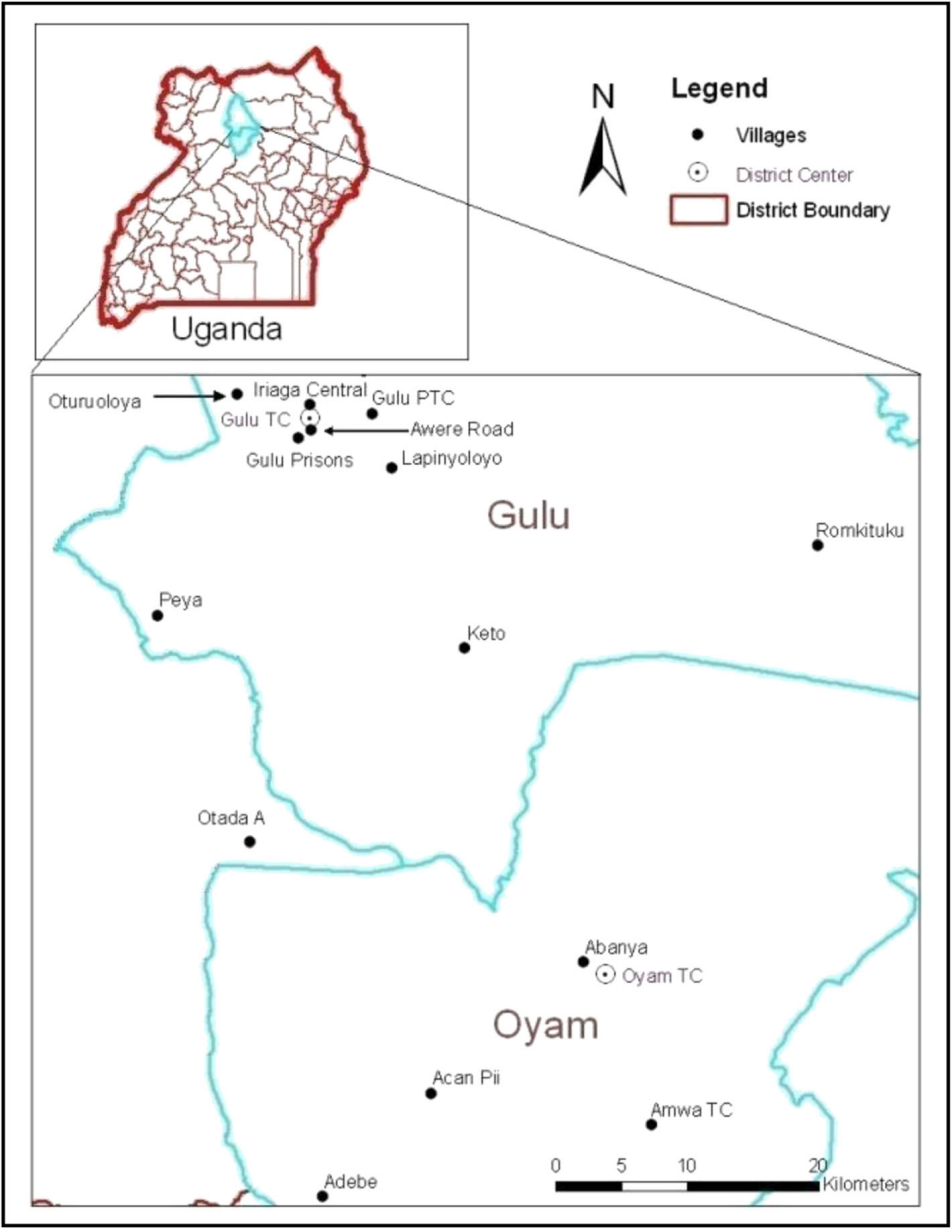
Study location and surveyed villages

To assess households’ perceived valuations of IRS programmes, survey enumerators conducted an in-person survey of households in the northern Ugandan districts of Gulu and Oyam (Fig. 1). These interviews, conducted in the local language, followed the single previous IRS round that had been conducted in each district. PMI’s IRS operations were typically conducted as follows:^1^ After advertising the programme through radio and consulting with village leaders and local health workers, the IRS team would arrive in the village at a designated time. Households were expected to have their homes unlocked with all belongings removed, and to have retrieved 10 l of water. IRS workers would then inspect the homes to ensure that they were empty, before dissolving a sachet of insecticide in their spray tanks using the water provided by the households. Spraying of the surfaces in the residential structures, usually consisting of one-to three-room mud huts, took less than one hour to complete. Spraying was typically conducted in the morning, and households were expected to remain outside of their homes for at least two hours. Households were given the choice of whether or not to participate in the IRS programme. Based on interviews with IRS workers and focus group participants, nonparticipation was uncommon. Reports suggested that nonparticipation usually occurred because household members were absent and their homes were locked (for unknown reasons).

### Choice experiment methods

To measure WTA we used a stated choice experiment. This survey-based method has respondents complete a number of choice tasks, in which they select the most-preferred option from a set of alternatives. In our choice experiment, the alternatives consisted of different types of IRS programmes, which varied according to the chemical used, the frequency of spraying, and reduction in malaria risk. Each task also included one alternative consisting of a one-time money payment in lieu of any IRS programme (with a correspondingly higher malaria risk for this alternative). WTA-based valuation measures for the different attributes of the IRS programmes can be estimated by analysing the frequency with which respondents select different alternatives, for different IRS configurations and different money amounts on offer across choice tasks. Similar stated choice methods have been used in a variety of public health and environmental contexts, including other mosquito control programmes in developed country contexts [29], as well as water and sanitation interventions in developing countries [30].

**Fig. 2:**
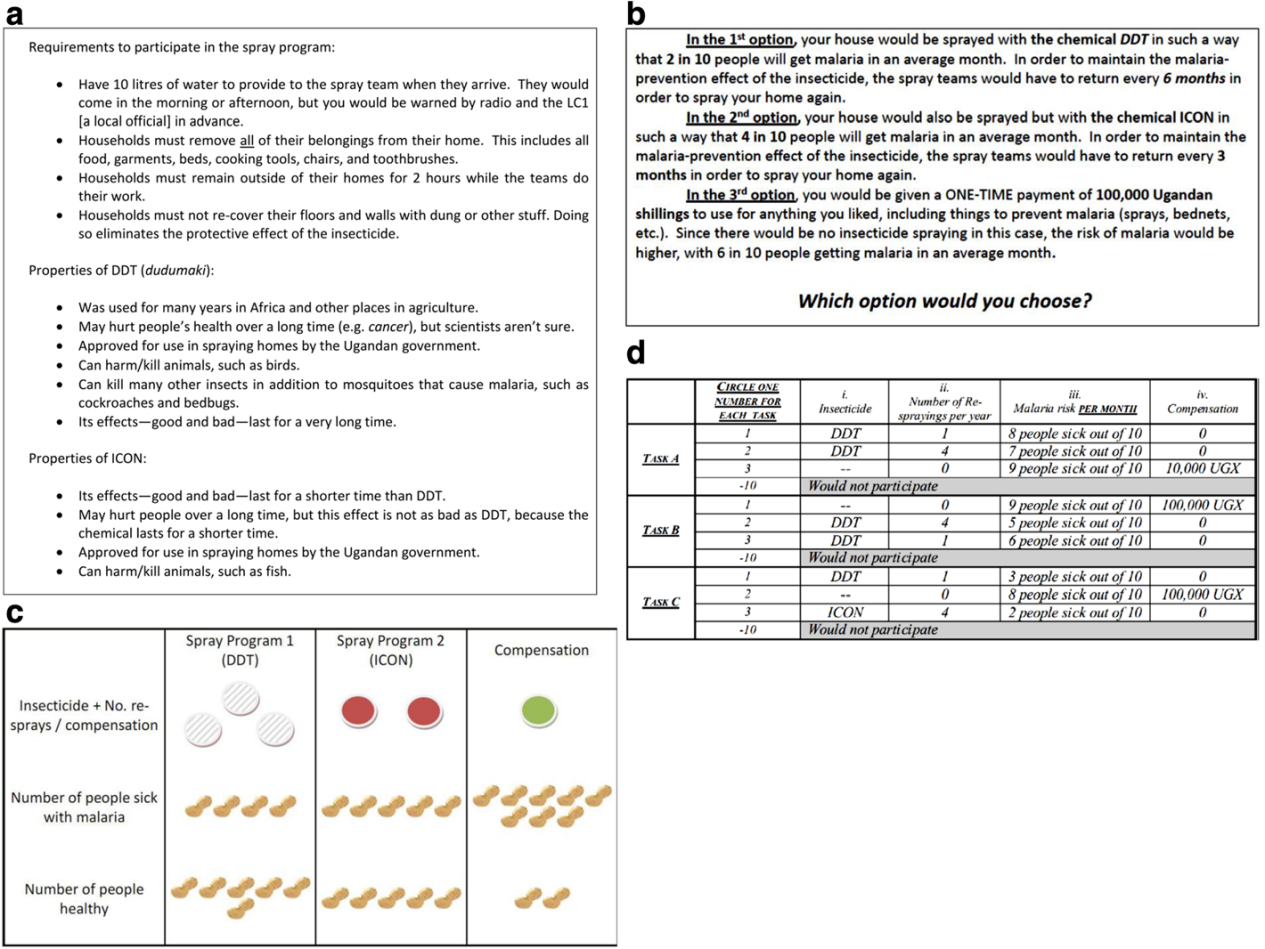
Choice experiment implementation. **a** information frame, **b** choice task script, **c** visual aid, **d** example choice task

The choice experiment format consisted of the survey interviewer first conveying to respondents a list of facts about IRS programmes and about the insecticides DDT and ICON (Fig. 2a). These were the only two insecticides that had been used in the region prior to the survey [28]. Focus group discussions indicated that many respondents could readily distinguish DDT, which had a much longer history in the region (e.g. having the local name of *dudumaki*), from ICON. IRS using DDT had also been the topic of a recent court injunction brought by local organic farmers, who were concerned that they would no longer be able to obtain organic certification–and the associated price premia–for products exported to Europe [31].

Interviewers then described the series of choice tasks the respondents would be asked to complete, using a set of visual aids to represent the attributes for the three alternatives in each choice task (Fig. 2b, c). Finally, respondents completed three separate tasks with different configurations of alternatives (Fig. 2d). The experimental design dictating the configuration of choice tasks and alternatives followed a D-optimal methodology with a prior based on the survey pretest [32].

### Econometric analysis

The data from the choice experiment were analysed with conditional and latent class logit econometric regression models. These models are based on the amount of “utility” a respondent derives from each alternative in a given choice task, such that each alternative’s total utility can be decomposed into the marginal utilities of its attributes [33]. In our case, the conditional logit model assumes that the utility respondent $i$ receives from alternative $j$ is:

1 $$ {V}_{ij} = {\beta}_0+{x}_j\beta +{\epsilon}_{ij} $$

where *x*_*j*_ is a vector of attributes for alternative *j*, *β* is a vector of marginal utilities defining preferences over programme attributes, and ${\epsilon}_{ij}$ is a random utility component akin to a standard regression error. For the choice experiment analysed here, the vectors of programme attributes and marginal utilities are:

2 $$ {x}_j=\left[\begin{array}{cc}\hfill \begin{array}{cc}\hfill ris{k}_j\hfill & \hfill mone{y}_j\hfill \end{array}\hfill & \hfill \begin{array}{cc}\hfill DD{T}_j\hfill & \hfill ICO{N}_j\hfill \end{array}\hfill \end{array}\right] $$

$$ \beta \hbox{'}=\left[\begin{array}{cc}\hfill \begin{array}{cc}\hfill {\beta}_{risk}\hfill & \hfill {\beta}_{money}\hfill \end{array}\hfill & \hfill \begin{array}{cc}\hfill {\beta}_{DDT}\hfill & \hfill {\beta}_{ICON}\hfill \end{array}\hfill \end{array}\right] $$

Each alternative’s attributes (*X*_*j*_) are defined as: malaria risk (*risk*_*j*_, ranging from a 0.1 to 0.9 probability of falling ill with malaria in a given month), the amount of money offered (*money*_*j*_), and the number of rounds of IRS per year (none, 1×, 2× or 4×) with each insecticide (*DDT*_*j*_ , *ICON*_*j*_). The corresponding *β* coefficients are the marginal utilities associated with these attributes.

Discrete choice models like the above employ the assumption that respondents select the alternative in a given choice task with the highest utility, including nonrandom and random components. This leads to a probabilistic statement of individual choice, with respondent $i$ selecting alternative $j$ from a given choice task $t$ with probability equal to:

3 $$ {p}_{\left(ij\Big|t\right)}= \Pr \left[{V}_{ij}\ge {V}_{ik}\Big|k\in t\right]= \Pr \left[{x}_j\beta -{x}_k\beta \ge {\epsilon}_{ik}-{\epsilon}_{ij}\Big|k\in t\right] $$

where *k* ∈ *t* is mathematical notation for “*k* belongs to *t*,” meaning in this case alternative *k* is in choice task *t*. Statistical estimation proceeds by making an assumption about the distribution of the random utility component *∈*_*ij*_. Most analyses assume, as we do here, the *∈*_*ij*_ to be identically and independently distributed according to a standardized Gumbel distribution [34], which leads to a conditional logit regression model for the choice probabilities ${p}_{\left(ij\Big|T\right)}=\frac{e^{x_j\beta }}{{\displaystyle {\sum}_{k\in t}}{e}^{x_k\beta }}$. These choice probabilities are then used to construct the likelihood of observing respondent *i*’s sequence of choices as:

4 $$ {L}_i\left(\beta \right)={\displaystyle \prod_{t\in {T}_i}}{\displaystyle \prod_{j\in t}}{p}_{\left(ij\Big|t\right)}^{c_{ij}}={\displaystyle \prod_{t\in {T}_i}}{\displaystyle \prod_{j\in t}}{\left[\frac{e^{x_j\beta }}{{\displaystyle {\sum}_{k\in t}}{e}^{x_k\beta }}\right]}^{c_{ijt}} $$

where *c*_*ijt*_ is a binary indicator for whether respondent *i* selected alternative *j* in choice task *t*, and *T*_*i*_ is the set of choice tasks completed by respondent *i*. The maximum likelihood estimate of the marginal utility vector is then obtained by maximizing with respect to *β* the sample log-likelihood function, defined as $\log \mathrm{\mathcal{L}}\left(\beta \right)={\displaystyle \sum_i} \log {L}_i\left(\beta \right)$.

After estimating *β*, monetary valuations of the DDT, ICON and malaria risk attributes are obtained by computing marginal WTA as the ratio between each marginal utility of these attributes and the marginal utility of money. For example, the term $WT{A}_{risk}=\left(10,\%\right)\times \frac{\beta_{\mathrm{risk}}}{\beta_{money}}$ provides an estimate of how much a respondent would have to be compensated in order to give up a permanent 10 % decrease in malaria risk.

The choice experiment asked respondents to compare permanent IRS programmes compared to a one-time compensatory payment (Fig. 2b). This framing was selected based on focus group discussions and pretesting. However, the policy community generally discusses risk reduction valuation in terms of annualized figures [35]. The annualized valuation formula for measuring the WTA a marginal change in attribute *k* over a period of one year is:

5 $$ \mathrm{Annual}\ WT{A}_k=\delta \times \frac{\beta_k}{\beta_{money}} $$

where *δ* is the effective annual discount rate for households in the population. The survey did not elicit a discount rate, and so we assume a rate of 10 %, based on a study by Bauer and Chytilová [36] measuring subjective discount rates in rural Ugandan households.

Eq. (1) assumes that every individual has the same marginal valuations when making choices about IRS participation. Yet it is possible reductions in average malaria risk are differentially valued across the population. For example some subpopulations may have higher exposure or experience greater consequences of malaria infection, relative to other groups. To examine whether some subgroups of respondents have higher valuations of IRS programmes, we also use a latent class logit model, to allow for different types of preferences in the population [34]. This method is commonly used in the environmental economics field [37]. The latent class choice model assumes discrete groups of preferences in the population: each latent class *l* is assumed to have a unique set of conditional logit regression coefficients *β* ^*l*^ defining preferences. In latent class models, class membership indicator variables are treated as missing data, and imputed via an expectation-maximization (EM) algorithm. If we observe that *i* ∈ *l*, then Eq. (5) implies that the likelihood of observing *i*’s sequence of choices is *L*_*i*_(*β* ^*l*^), meaning that we could easily estimate these marginal utilities using a conditional logit model for each class. Furthermore, we could estimate a probability model to predict class membership based on a vector of respondent-specific covariates *Z*_*i*_ (e.g. socioeconomic characteristics). The standard model used to predict class membership is a multinomial logit model:

6 $$ {\pi}_{i,l}\left(\gamma \right)=\frac{e^{Z_i{\gamma}_l}}{{\displaystyle {\sum}_{\iota }}{e}^{Z_i{\gamma}_{\iota }}} $$

where *γ* = (*γ*_1_, …, *γ*_*L*_)’ is the collection of coefficient vectors (one for each class) defining the marginal effect of each variable in *Z*_*i*_ on the prior log-odds of belonging to class *l*.

However, because we do not observe class membership, we impute it via Bayes’ formula. Given a prior probability *π*_*i*,*l*_ that *i* ∈ *l*, then the posterior probability that *i* ∈ *l* is:

7 $$ {\rho}_{i,l}=\frac{\pi_{i,l}{L}_i\left({\beta}^l\right)}{{\displaystyle {\sum}_{\iota }}{\pi}_{i,\iota }{L}_i\left({\beta}^{\iota}\right)} $$

The EM algorithm proceeds by taking some initial guesses for the individual posterior class membership probabilities *ρ*_*i*,*l*_, and using these to estimate the marginal utilities *β* ^*l*^ and the unconditional class probabilities *π*_*i*,*l*_ by maximizing the *expected* log-likelihood function for the sample, defined as:

8 $$ \mathbb{E}\left[ \log \mathrm{\mathcal{L}}\left(\beta, \gamma \right)\right]={\displaystyle \sum_l}{\displaystyle \sum_i}{\rho}_{i,l}\left[ \log {L}_i\left({\beta}^l\right)+ \log {\pi}_{i,l}\left(\gamma \right)\right] $$

In this paper we assume two latent classes, the maximum number that could be estimated with the available data. The relevant estimates obtained from the latent class model are the WTA estimates for each attribute in the choice experiment for each latent class, as well as the marginal effects of respondent-specific characteristics on (a) prior class membership probabilities (as in Eq. 6) and (b) on WTA estimates for malaria risk reductions and other attributes in the experiment. Details on how these marginal effects and their standard errors were obtained, as well as issues related to survey sampling weights, are reported in the Additional file 2.

### Survey data collection

The survey data were collected through a three-step process consisting of focus group discussions (FGDs); survey drafting, translation and pre-testing; and sampling of households and administration of the final questionnaire. Six FGDs were conducted in Gulu town, Uganda, and emphasized discussion of the perceived burden of malaria and the relative benefit of different control methods, as well as a discussion of general risks faced by households. A structured questionnaire was then drafted and translated into the local Acholi language, based on transcripts from the FGDs. This questionnaire was pre-tested and revised around the Gulu town for two weeks in October 2009. The final questionnaire was back-translated by an independent party to ensure accuracy of the content.

Households were sampled for participation in the survey so as to permit statistical inference on the populations of Gulu and Oyam districts of northern Uganda (Fig. 1). A three-stage clustered sampling design was employed. Parishes (administrative units within each district) were selected with probability proportional to size (PPS), using population projections from the Ugandan Bureau of Statistics (UBOS). The sampled parishes were stratified so that nine parishes were sampled with PPS in Gulu and six in Oyam. One village was selected at random from each parish using lists from the districts’ headquarters, and 40 households were randomly selected for the survey within each sampled village. This sampling procedure requires the use of sampling weights in the statistical analysis [38], which are employed throughout the statistical analysis whenever feasible.

Statistical and econometric analysis of the data was performed using Stata/IC® Version 12.1 from StataCorp. All 612 respondents sampled for the survey gave their informed consent to be interviewed; 588 (96 %) questionnaires were sufficiently complete for use in this study. Fifty-eight percent of surveyed respondents were heads of their households, 38 % were spouses of the household head, and the remaining 4 % were other relatives of the household head.

## Results and discussion

General socioeconomic and malaria-specific characteristics of the sampled households, as well as the individual respondents, are presented in Table 2. The sample appears representative of the population of Gulu and Oyam districts in 2009, based on limited data from other externally reported statistics for this region and time period that were available (last column of Table 2). The estimated mean household size, at six members, in the survey sample is around one member greater than the same statistic (at five members) reported in the 2009 Malaria Indicators Survey (MIS) [39]. The mean age of members and number of children under 10 in a given household is approximately the same across the survey sample and the MIS. The survey sample appears less educated than the most relevant comparable statistic we could find in the Demographic Health Survey (DHS) [40], with 11 % of individuals over six years of age covered by the survey having completed some secondary education, as compared to a comparable statistic of 16 % in the DHS.

The survey data pertaining to malaria burden prevention roughly agree with available external statistics. The survey data indicate a high malaria burden during the time period of the study. The malaria incidence measures in Table 1 indicate that approximately one in five adults and one in four children under ten had been diagnosed with malaria in the past month, at a health clinic based on a blood test. While independent sources do not provide comparable statistics for malaria burden in this region and time period, the 2009 MIS does confirm an extremely high burden of malaria in the mid-northern region (including Gulu and Oyam) for this time period, reporting 80 % malaria prevalence among children under 5 years of age based on data from rapid diagnostic testing (Table 6.3) [39]. Ownership of mosquito nets is also very similar between the survey and the 2009 MIS.

**Table 1:** Description of choice experiment attributes and levels

Attribute	Description	Levels and values
Malaria risk	Average fraction of people out of 10 getting sick with malaria in an average month.	1/10 to 9/10, increments of 1/10
Compensation	One-time payment offered to respondent (in place of IRS).^a^	$0, $4, $22, $43, $65, $217
DDT	Frequency that DDT is sprayed (for IRS programmes)	0,1,2, or 4 times per year
ICON	Frequency that ICON is sprayed (for IRS programmes), mutually exclusive with DDT.	0,1,2, or 4 times per year

*Notes:* ^a^Compensation amounts were described to respondents in local currency (Ugandan shillings), but are presented here in USD 2009 for ease of interpretation

The largest and most relevant discrepancy between the survey data and external statistics pertains to IRS participation. The survey data indicate an 80 % participation rate in IRS, whereas the only comparable external statistic that could be found is from the United States President’s Malaria Initiative (pp.) [19, 28], which reports a participation rate of 93 % in its pilot round of IRS using DDT in Oyam district prior to survey data collection. (Subsequent rounds of IRS, occurring after the survey, reported participation rates above 99 %, [41].) Discrepancies between the survey sample and externally reported statistics could be due to measurement error in both the survey data and the externally reported statistics, as well as imperfect comparison subpopulations in the external statistics. The MIS, for example, only reports figures for the ‘Mid Northern’ region of Uganda, not for Gulu and Oyam specifically, and a similar issue applies for the 2011 DHS, which only reports statistics for the ‘North’ (and for data collected two years after the survey analysed here).

Turning to the summary statistics of the choice experiment results, Table 3 reports general choice patterns in the experiment, and Fig. 2 visually shows how respondents resolved the tradeoff between foregone monetary compensation and reduced malaria risk. Respondents expressed a strong favourable preference for IRS, selecting one of the two IRS alternatives available in each choice task over money compensation 81 % of the time (Table 3), and 72 % of respondents always selected an IRS alternative in all three of their choice tasks.^2^ Respondents did not appear to favour one chemical over another, selecting DDT in almost exactly half of the tasks in which an IRS alternative was selected and opting for ICON in the remainder.

Respondents’ preferences and choices do appear sensitive to the scale of potential malaria risk reduction, as well as the potential amount of money on offer. Table 2 shows that while respondents strongly preferred IRS, they did not always select the alternative yielding the lowest malaria risk (with only 31 % of cases exhibiting this behaviour). Furthermore, 8 % of respondents always chose the money alternative over IRS. This is a statistically significant deviation (*P*-value < 1 %) from the 3.7 % frequency with which such behaviour would be observed by random chance (0.037 = (1/3)^3^). Additionally, a group of respondents always chose monetary compensation instead of IRS in 10 % of choice tasks.

**Table 2:** Summary statistics

Variable	Mean/Frequency	Standard Dev.	External statistic^5^
Number of households surveyed	588		
*Household-level variables*			
Household size (members)	6.1	2.6	4.9^5(a)^
Average household age (years)	21	11	20^5(a)^
Number of children under 10	2.1	1.6	1.8^5(a)^
Value of household assets	$266	$459	
Monthly household income	$44	$71	
Education ≥ some secondary^1^	11.2 %	--	16 %^5(b)^
Monthly malaria incidence^1,2^			
Total population	0.17	0.24	
Children under 10	0.24	0.35	
Participated in previous IRS			
Cluster weights only	80 %	--	93 %^5(c)^
Cluster & household weights^1^	83 %	--	
Mosquito nets			
per household	1.6	1.8	1.4^5(a)^
per person^1,3^	0.26	0.35	
*Respondent-level variables*			
Age	39	15	
Female	54 %	--	
Education $\ge$ some secondary	27 %	--	
Ill with malaria in past month?^2^	13 %	--	
Perceived malaria risk^4^	0.33	0.22	
*Respondent believes…*			
Mosquitoes cause malaria^1^	90 %	--	
Standing water predicts mosquito abundance^1^	54 %	--	
IRS is effective^1^	82 %	--	

*Notes:* Statistics calculated using sampling weights adjusting for cluster sampling, unless otherwise noted. *1.* Sampling weights accounting for both cluster sampling and household size used for these statistics, *2.* Malaria diagnosis measures are self-reports indicating whether each member in the household was diagnosed with malaria in the past month by going “to the health facility where they took blood,” so this incidence measure is an underestimate of actual malaria incidence in the population at the time of study. Other, less conservative measures are reported in the survey, but are not shown here. *3.* Refers to both insecticide-treated or untreated, *4.* Measured as respondent subjective expectation of how many people out of 10 would fall ill with malaria in next 30 days, *5.* External data-sources used for comparison: *(a)* MIS [39], for ‘Mid Northern’ subpopulation where available, otherwise ‘Rural,’ (*b)* DHS [40], for ‘North’ subpopulation, *(c)* PMI [48]

**Fig. 3:**
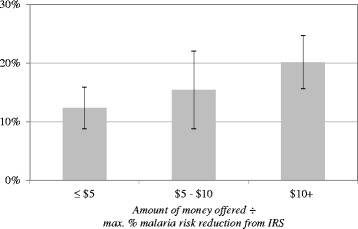
Percent of choice tasks in which money selected over IRS, by cost-effectiveness ratio. Error bars show 95 % confidence interval of mean estimate of 1 686 choice tasks distributed across 588 respondents. Sample weights used in computation

Figure 3 shows that, as opting for IRS over money compensation represents a more significant opportunity cost, respondents are more likely to select the money alternative. This figure plots the frequency with which respondents selected money over IRS, for different cost-effectiveness ratios of IRS: the cost-effectiveness ratio here is defined as the amount of money offered in the compensation alternative in each choice task divided by the maximum, permanent malaria risk reduction (expressed as percent probability) that could be obtained from either IRS alternative in that task. The figure shows that when the amount of money offered is less than $5 per 1 % of foregone risk reduction, respondents only select the money alternative 12.3 % of the time, whereas this frequency increases to 20.2 % when the tradeoff climbs to greater than $10 per 1.0 % of foregone risk reduction. A probit regression using the cost-effectiveness ratio to predict the probability that respondents select the money alternative over IRS implies that this ratio is a statistically significant predictor of behaviour (*P*-value = 0.006, regression results presented in Additional file 2). If we view the cost-effectiveness ratio as a price of malaria risk reduction (in terms of the marginal opportunity cost of giving up money to obtain the risk reduction), then this regression implies a ‘price elasticity of demand’ of approximately 0.15, meaning that a 1 % increase in this ratio decreases demand for IRS by 0.14 % (this figure is based on a marginal effects computation using the probit regression, shown in the Additional file 2).

While the above results provide useful descriptive analysis of the behaviour observed in the choice experiment, econometric models described in the previous section are necessary to disentangle the partial effects of each attribute considered in the IRS alternatives, as well as to identify the effect of respondent socioeconomic and malaria-specific characteristics on WTA valuations of malaria risk reductions. Table 3 shows the estimated WTA for specific attributes of IRS. Results from the conditional logit regression model, corresponding to Eq. (1) above, are presented in the first two columns of numbers in terms of WTA, i.e. the ratio of each IRS attribute’s regression coefficient divided by the regression coefficient on the money alternative (the raw regression coefficients do not have easily interpretable units and so are shown in the Additional file 2).

**Table 3:** Summary outcomes from the choice experiment

	Frequency	95 % Confidence Interval
IRS alternative selected^a^	82 %	(81 %–87 %)
using DDT	42 %	(39 %–46 %)
using lambda-cyhalothrin (ICON)	40 %	(38 %–44 %)
Always selected^b^		
Money alternative	8 %	(6 %–11 %)
IRS alternative	74 %	(70 %–78 %)
DDT alternative	13 %	(9 %–16 %)
ICON alternative	10 %	(7 %–13 %)
Lowest malaria risk alternative	31 %	(26 %–35 %)

*Notes:* Sampling weights applied to account for cluster sampling. ^a^ Percentage of choice tasks in sample (3 per respondent). ^b^ Percentage of survey respondents exhibiting one of the listed behavioural patterns

The results in Table 3 indicate a significantly positive mean WTA for the malaria reduction benefits of IRS, equating to an annualized valuation of approximately $9 per 10 % malaria risk reduction. Malaria reduction benefits are evidently not the only significant benefits of IRS, as perceived by respondents. The mean WTA valuations of a round of DDT and ICON are $56 and $54 respectively (with no significant difference in WTA between the chemicals). These valuations are independent of the malaria reduction benefits of IRS. These side effects of IRS are equivalent to a malaria risk reduction of roughly 60 %.

The last two columns of Table 4 show results of the latent class logit model, which allows for respondent heterogeneity in WTA. This regression model reveals two types of preferences among the surveyed respondents. One type of preference (approximately 80 % of the sample) corresponds to a relatively high valuation for all aspects of IRS, including both malaria reduction benefits and side effects. For these respondents, the side benefits of IRS appear to be equivalent to between a 40–50 % reduction in malaria risk.

**Table 4:** Estimated annual willingness to accept (WTA)

	Conditional logit model^a^		Latent class logit model^b^			
			Class 1 (High WTA)		Class 2 (Low WTA)	
*Alternative-specific attributes*	*WTA*	*Std. Err.*	*WTA*	*Std. Err.*	*WTA*	*Std. Err.*
Foregone 10 % malaria risk reduction	$8.94***	($3.40)	$19.35***	($5.35)	$0.38	($0.31)
*One IRS round foregone*						
DDT-based	$56.38***	($14.57)	$87.53***	($20.43)	-$1.97***	($0.94)
ICON-based	$53.78***	($13.69)	$84.32***	($20.01)	-$2.79***	($1.07)
*Predicted class sizes*						
Unconditional			80 %		20 %	
(w/ sample weights)			82 %		18 %	
Conditional			81 %		19 %	
(w/ sample weights)			84 %		16 %	
Respondents	588		588			
Choice tasks per respondent	3		3			
Model degrees of freedom	4		25			
Log-likelihood	−1 376		−1 166			

*Notes:* ***, ** and * indicate statistical significance at the 1, 5 and 10 % levels, respectively. Computations based on conditional and latent class logit model estimates (Additional file 2: Table A1). A 10 % discount rate is applied to convert the choice model coefficients to annual WTA, according to estimates reported by Bauer and Chytilová [36]. Dollar values in 2009 USD. Standard errors calculated clustering at the respondent level (i.e. across choice tasks). ^a^ Model estimated with sampling weights. Model estimated without sampling weights yields similar results, but with a 34 % lower (in magnitude) malaria risk WTA and a log-likelihood value of −1419. ^b^ Model and reported log-likelihood first estimated without sampling weights, due to software limitations. To account for sampling design, sampling weights applied to class membership model and imputed class sizes reported here with and without sample weights

The other type of preference (the remaining 20 % of respondents) corresponds to a relatively low valuation for malaria reduction, and in fact a negative estimated value of IRS side effects–implying a nonmonetary cost of IRS participation for these individuals. The choice experiment results for this subgroup suggest, for example, that IRS programmes consisting of two rounds per year would need to achieve between a 10–15 % reduction in malaria risk in order to yield a net positive value for these individuals.

The finding that one group had a very high, positive valuation of IRS, whereas the other group had a negative valuation of the side effects, conforms with the FGDs and anecdotal reports by the survey enumerators that opinions about IRS were polarized. Some respondents were strongly positive about IRS programmes, not only for malaria reduction but also for their perceived sanitizing effects (e.g. the killing of nuisance insects). Other respondents reported experiencing negative health effects following treatment of their home with IRS.

The effect of covariates on IRS valuation can be estimated in the latent class model, by including how respondent characteristics predict the probability of belonging to the high- or low-WTA groups. As shown in Table 5, the statistically significant factors positively influencing the likelihood of belonging to the high-risk group are mosquito net ownership, whether the respondent believes that standing water is a predictor of mosquito abundance, as well as whether the respondent in fact believes IRS is effective at reducing malaria risk (even though the choice experiment clearly defined the expected malaria risk reduction for each IRS alternative). The statistically significant factors positively affecting the likelihood of belonging to the low-WTA group are the average age of members in the respondent’s household, and whether the respondent is female. Income and wealth measures from the survey are not statistically significant predictors of WTA. While this would be a surprising finding if we were measuring WTP, it is less surprising that these variables are not associated with WTA, since the latter is not bounded by budget constraints.

**Table 5:** Marginal effects of household and respondent covariates on IRS preferences

	Prob. in high-WTA group		Marginal effect on expected WTA to forego:		
	Marginal effect	Std. Err.	10 % decrease in malaria risk	One round of DDT	One round of ICON
*Household-level variables*					
Household size (members)	+0.39 %	(0.706 %)	+$0.07	+$0.35	+$0.34
Average household age (years)	−0.34 %*	(0.191 %)	-$0.06	-$0.30	-$0.29
Number of children under 10	−0.93 %	(1.309 %)	-$0.18	-$0.83	-$0.81
Value of household assets	−0.01 %	(0.004 %)	-$0.00	-$0.01	-$0.01
Monthly household income	+0.05 %	(0.038 %)	+$0.01	+$0.04	+$0.04
Monthly malaria incidence	−4.06 %	(7.526 %)	-$0.77	-$3.63	-$3.54
Participated in previous IRS^a^	+8.53 %	(7.210 %)	+$1.62	+$7.63	+$7.43
Mosquito nets per person	+9.70 %**	(4.862 %)	+$1.84	+$8.68	+$8.45
*Respondent-level variables*					
Age	+0.11 %	(0.132 %)	+$0.02	+$0.10	+$0.10
Female^1^	−5.71 %**	(2.916 %)	-$1.08	-$5.11	-$4.97
Education $$ \ge $$ some secondary^a^	−6.26 %	(4.423 %)	-$1.19	-$5.61	-$5.46
Ill with malaria in past month?^a^	+5.05 %	(3.886 %)	+$0.96	+$4.52	+$4.40
Perceived malaria risk	+11.10 %	(9.599 %)	+$2.10	+$9.93	+$9.67
*Respondent believes:* ^a^					
Mosquitoes cause malaria	+3.56 %	(5.677 %)	+$0.68	+$3.19	+$3.10
Standing water predicts mosquito abundance	+7.56 %***	(2.787 %)	+$1.43	+$6.77	+$6.59
IRS is effective	+19.45 %***	(6.897 %)	+$3.69	+$17.41	+$16.95

*Notes:* Estimated covariate effects from the latent class logit model (Table 4 and Additional file 2: Table A1). ***, ** and * indicate statistical significance at the 1, 5 and 10 % levels, respectively. For WTA calculations, 10 % discount rate is applied to convert the choice model coefficients to annual WTA [36]. ^a^ Marginal effect calculated for discrete change in binary variable

In terms of the effects of these covariates on monetary valuations of IRS programme attributes, we can see for example in Table 5 that a household owning an additional mosquito net is associated with a $1.84 addition to the respondent’s WTA valuation of a 10 % malaria risk reduction. An important caveat about this finding is that while Table 5 shows which respondent characteristics are associated with higher or lower WTA, we cannot infer from our data *why* these characteristics statistically predict WTA. For example, mosquito nets may in fact be a malaria prevention substitute for IRS, which would lead one to expect that owning a mosquito net lowers the value of IRS. However, households owning a mosquito net may be a priori more concerned about malaria, in which case this variable would be serving as a proxy for individuals who place greater value on malaria risk reductions.

## Conclusions

This study shows that a large majority of the population in a region with high, endemic malaria and exposure to public IRS programmes places a high value on such programmes, due both to the malaria reductions benefits of these programmes and to perceived beneficial side effects. However, a significant minority–approximately one in five household heads–perceives significantly lower value from these programmes, to the point of experiencing a net cost from the side effects of IRS.

These results are important when considering how to sustain public support for IRS and other vector control programmes. While our finding of a high WTA among a majority of respondents suggests that IRS programmes do enjoy widespread support in this region, the 20 % of respondents placing a relatively lower value on IRS is not an insignificant portion of the population, particularly when considering the extremely high malaria burden observed in our survey as well as in other data from this region and time period [39, 42]. Understanding the reasons underlying these low valuations is important to avoid adverse public reactions imposing constraints that might limit the ability of programme managers to flexibly respond to future biological dynamics, such as the evolution of insecticide resistance.

Our results suggest that higher values for IRS programmes are harboured by those who are better informed about factors determining malaria transmission (for example, whether standing water is a predictor of mosquito abundance), and by those who have taken prior preventative action to avoid malaria, like purchasing mosquito nets or in fact participating in a previous IRS round. In this way our study relates to other research demonstrating the importance of knowledge (particularly maternal knowledge) about malaria, in participating in malaria prevention programmes [43, 44]. This body of research suggests that increasing knowledge and awareness about malaria transmission and potential preventative options can have a measureable impact on behaviour.

Our results are also relevant for considering possible economic instruments for increasing adoption of malaria prevention practices and technologies. As has been well-studied in the area of vaccination [45, 46], an economic argument can be made for subsidizing individual efforts to prevent an infectious disease, because such individual efforts, when aggregated, have positive spillovers on the population as a whole. By econometrically estimating a preference-based model of prevention choices as we do here, we can move closer to the goal of estimating socially optimal subsidies for malaria prevention efforts (subsidies which may go beyond free provision of IRS in our case, due to nonmonetary side effects). This objective of translating such a theory to measurement would require fusion of economic evaluation with epidemiological modelling of the direct and indirect malaria reduction benefits of IRS and other vector control efforts. For example, such an epidemiological model would be necessary to model the effects of IRS participation on individual risk and population-level malaria incidence [47]. This broader agenda comprises a relevant topic for future research.

There are two significant limitations we acknowledge in this study. First, our data come from a single survey round and thus represent only a snapshot of preferences related to IRS. Such preferences may evolve over time in particular because of households’ increased experience with these programmes and other vector control alternatives, as well as in response to the non-marginal changes in malaria risk that are occurring from the continued scale-up of vector control in combination with improved case management (e.g. the use of rapid diagnostic tests in combination with current generation antimalarial drugs). This limitation motivates repeating similar studies in the future to systematically monitor public support for these programmes over time. Another limitation is that our choice experiment obtained WTA for a permanent IRS programme, whereas an annual valuation measure is more practical. Consequently, we must rely on a 10 % discount rate reported by Bauer and Chytilová [36] to convert our estimates to annual terms. However, this limitation can be partially addressed by noting that the discount rate only affects the absolute dollar values reported in this study, but does not affect the relative values, for example between malaria risk reductions and IRS side effects.

It is important to note in closing that the intent of this analysis is more nuanced than simply arguing for or against IRS programmes on the basis of public attitudes. Rather, our aim is to quantitatively assess how the different components of IRS programmes shape preferences. By doing so, we argue that such programmes can be more completely evaluated from the perspective of the households who have the most at stake in their deployment.

## Additional files

Additional file 1: Multilingual abstracts in the five official working languages of the United Nations. (PDF 560 kb)

Additional file 2: Supplementary material. (PDF 341 kb)

Additional file 3: Understanding households’ preferences regarding indoor residual spraying, malaria-related risks, and other risks. (PDF 1056 kb)

## Abbreviations

DDT: Dichlorodiphenyltrichloroethane
DHS: Demographic Health Survey
FGD: Focus group discussion
IRS: Indoor residual spraying
ITN: Insecticide-treated net
MIS: Malaria Indicators Survey
NMCP: National malaria control program
PMI: President’s Malaria Initiative (US Government)
PPS: Probability proportional to size
UBOS: Ugandan Bureau of Statistics
WTA: Willingness to accept
WTP: Willingness to pay

## Footnotes

1 This background material is based on personal communication with multiple officials involved with PMI’s Gulu and Oyam IRS operations in 2008 and 2009.

2 None of the respondents opted for the status quo in any of the choice tasks, which is not surprising given that some money compensation was available in every task: selecting the status quo would have amounted to leaving money on the table.
