# Supplementary material for: Household perceptions and subjective valuations of indoor residual spraying programmes to control malaria in northern Uganda
Source: Infect Dis Poverty. 2016 Oct 6;5:100. doi: 10.1186/s40249-016-0190-1 (PMC5053089; doi:10.1186/s40249-016-0190-1)
Supplement: Supplementary file 1 — Multilingual abstracts in the five official working languages of the United Nations. (PDF 560 kb) [file 40249_2016_190_MOESM1_ESM.pdf]

Translation of the abstract into the five official working languages of the United Nations

تصورات الأسر وتقديراتها الشخصية لبرامج الرش الموضعي للأماكن المغلقة لمكافحة الملاريا في شمال أوغندا

زاكاري س. براون، راندال أ. كريمر، ديفيد أوكان، كريستين أورييما

ملخص

خلفية: لا تزال الأدوات التي تعتمد على المبيدات الحشرية حيوية لمكافحة الأمراض التي تنقلها الحشرات في أوغندا. تأمين الدعم الشعبي من السكان المستهدفين لهذه الأدوات هو عنصر هام في الحفاظ على فعاليتها على المدى الطويل. إلا أنه لا توجد سوى القليل من الأدلة المتاحة للفوائد والتكاليف المتوقعة من برامج مكافحة ناقلات الأمراض بين الأسر المستهدفة.

الطرق: تم إجراء مسح لعينة عشوائية عنقودية من 612 أسرة في منطقتي جولو وأويام في شمال أوغندا خلال فترة كان فيها معدل انتقال الملاريا عال جدا حيث تم تطبيق برنامج تجريبي للرش الموضعي داخل الأماكن المغلقة (IRS). أجريت تجربة اختيار منفصلة داخل المسح، حيث اختار المشاركون برامج الرش الموضعي للأماكن المغلقة المختلفة وفقا للتعويضات المادية في سلسلة متحكم فيها بشكل تجريبي، ضمن مجموعات اختيار افتراضي. وقد تم تحليل البيانات باستخدام نماذج الانحدار اللوغاريتمي المشروطة لتقدير الاستعداد للقبول (WTA) نظير مبلغ تعويض مالي مقابل تخفيض خطر الإصابة بالملاريا. واستخدمت نماذج الصنف الكامن لتحليل ما إذا كانت الخصائص المشترك تنبئ بالاستعداد للقبول.

النتائج: يقدر متوسط الاستعداد للقبول بـ8.94 دولار سنويا لتخفيض خطر الملاريا بنسبة 10٪، وقدرت المنافع المشتركة إضافية من برامج الرش الموضعي للأماكن المغلقة أن تكون قيمتها في المتوسط 54 دولار - 56 دولار (اعتمادا على نوع الحشرات) في الجولة من برامج الرش الموضعي للأماكن المغلقة. ويلاحظ عدم تجانس كبير: أربعة من كل خمسة أرباب أسر في شمال أوغندا أعطوا تقييمات عالية لبرامج برامج الرش الموضعي للأماكن المغلقة، في حين أن 20٪ الباقين عانوا من آثار جانبية مكلفة لبرامج الرش الموضعي للأماكن المغلقة (تبلغ قيمتها بين دولارين و 3 دولارات في كل جولة). وشمل تنبؤ ذا دلالة إحصائية للانتماء إلى الجماعة ذات القيمة العالية جنس المشترك، سن أفراد الأسرة، المشاركة السابقة في برامج الرش الموضعي للأماكن المغلقة، المعرفة الأساسية بتكاثر البعوض، عدد الناموسيات التي تملكها الأسرة. لم يتم العثور على بدائل لدخل الأسرة والثروة يمكن أن تكون عوامل تنبؤ ذات دلالة إحصائية للاستعداد للقبول.

الاستنتاجات: تشير هذه الدراسة إلى أن غالبية الناس في مناطق انتقال الملاريا بمعدلات عالية مثل شمال أوغندا ذات قيمة عالية في برامج مكافحة النواقل باستخدام برامج الرش الموضعي للأماكن المغلقة. ومع ذلك، هناك تباين كبير من حيث الآثار الجانبية الملحوظة (الإيجابية والسلبية). وهي لها آثار للحفاظ على الدعم الشعبي لهذه البرامج على المدى الطويل.

Translated from English version into Arabic by Mahmoud Sami, through

TRANSLATORS WITHOUT BORDERS

乌干达北部地区采用室内滞留喷洒项目进行疟疾防治的家庭观点和主观评价

Zachary S. Brown, Randall A. Kramer, David Ocan, Christine Oryema

摘要

引言：在乌干达，以杀虫剂为基础进行媒介传染病控制依然是主要的控制手段。从目标人群获得公众支持是维持其长期效果的重要组成部分。然而，关于目标人群媒介控制项目的感知利益尚缺乏定量实证。

方法：在乌干达北部古卢和奥亚姆地区疟疾高传播时期开展一项室内滞留喷洒(IRS)项目，

并采取随机集群抽样对 612 个家庭进行调查。在调查中采用离散选择试验，应答者在一系列受控的，包括多项选择集的实验中针对与金钱补偿相关的不同 IRS 项目表明他们的偏好。数据结果采用条件逻辑回归模型计算应答者接受一定数量的金钱补偿的意愿（WTA）来代替先前的疟疾风险降低。采用潜类别模型来分析是否应答者的特征能够预测 WTA。

结果：平均接受意愿（WTA）为每年$8.94 来降低 10%的疟疾风险，IRS 额外的协同效益为平均每轮 IRS $54-$56（取决于杀虫剂类型）。项目中存在显著地异质性：北乌干达 4/5 的户主对 IRS 具有较高的评价，剩余的 20%户主却经历着成本较高的 IRS 副作用（每轮$2-3$之间）。统计上属于高估值群体的预测指标包括应答者的性别、家庭成员平均年龄、参与过之前 IRS 项目、蚊虫繁殖的基本知识以及拥有的蚊帐数目。家庭收入和财富指标与 WTA 无显著相关性。

结论：本研究表明乌干达北部疟疾高流行区的大多数人群认同在媒介控制项目中采用 IRS。然而，在 IRS 副作用认识方面（包括正面和负面的）有显著的异质性。这将对于获取公众对这些项目的长期支持具有一定意义。

Translated from English version into Chinese by Xin-Yu Feng, edited by Pin Yang

Perceptions des ménages et évaluation subjective des programmes de pulvérisation à effet rémanent à l'intérieur dans la lutte contre le paludisme dans le nord de l'Ouganda

Zachary S. Brown, Randall A. Kramer, David Ocan, Christine Oryema

Résumé

Contexte : les outils reposant sur l'utilisation d'insecticide restent essentiels à la lutte contre des maladies à transmission vectorielle en Ouganda. L'obtention du soutien public de la part de populations ciblées à l'égard de tels outils assure dans une large mesure la pérennité de leur efficacité à long terme. Il n'existe néanmoins que peu de données quantitatives relatives à la perception des avantages et des coûts de programmes de lutte antivectorielle parmi les ménages ciblés.

Méthodes : une enquête a été réalisée auprès d'un échantillon aléatoire en grappes de 612 ménages dans les districts de Gulu et Oyam dans le nord de l'Ouganda au cours d'une période caractérisée par un degré très élevé de transmission du paludisme et suite à un programme pilote de pulvérisation à effet rémanent à l'intérieur (IRS). Une expérimentation discrète des choix a été menée dans le cadre de l'enquête et nécessitait que les personnes interrogées indiquent leurs préférences à l'égard de différents programmes d'IRS par rapport à la compensation financière dans plusieurs séries de choix hypothétiques fondées sur l'expérimentation. Les données ont été analysées à l'aide de modèles de régression logit conditionnel afin d'estimer la disposition des personnes interrogées à accepter une certaine somme d'argent à titre de compensation de l'abandon de mesures de réduction du risque de paludisme. Des modèles de structure latente ont été utilisés afin d'analyser si les caractéristiques des personnes interrogées permettaient de prédire leur disposition.

Résultats : la disposition moyenne est estimée à 8,94 dollars US par an pour une réduction de 10 % du risque de paludisme et les avantages concomitants supplémentaires de l'IRS étaient estimés en moyenne de 54 à 56 dollars US (en fonction du type d'insecticide) par cycle d'IRS. Une hétérogénéité significative est observée : quatre chefs de ménage sur cinq dans le nord de l'Ouganda évaluent très positivement les programmes d'IRS, tandis que les 20 % restants font face à d'onéreux effets secondaires de l'IRS (estimés de 2 à 3 dollars par cycle). Les prédicteurs significatifs d'un point de vue statistique relatifs à l'appartenance au groupe accordant une grande valeur aux programmes d'IRS comprennent le sexe, l'âge moyen des membres du ménage, la participation à des programmes d'IRS antérieurs, les connaissances de base relatives à la reproduction des moustiques et le nombre de moustiquaires que le ménage détient. Les indicateurs pour le revenu et la richesse des ménages ne peuvent pas être considérés comme des prédicteurs significatifs d'un point de vue statistique de la disposition.
Conclusions : cette étude suggère que la majorité des personnes vivant dans des zones à taux élevé de transmission de paludisme comme le nord de l'Ouganda accordent une grande importance aux programmes de contrôle des vecteurs, tels que l'IRS. Nous constatons néanmoins une hétérogénéité significative en termes de perception des effets secondaires (positifs et négatifs), ce qui a des conséquences sur la pérennité du soutien public en faveur de ces programmes à long terme.

Translated from English version into French by Suzanne Assenat, through

TRANSLATORS
WITHOUT BORDERS

Бытовые представления и субъективные оценки программы распыления инсектицидов остаточного действия внутри помещений с целью контроля малярии в северной части Уганды

Zachary S. Brown, Randall A. Kramer, David Ocan, Christine Oryema

Аннотация

Справочная информация: Средства борьбы с насекомыми до сих пор являются критическими инструментами в борьбе с трансмиссивными заболеваниями в Уганде. Обеспечение общественной поддержки со стороны целевых групп населения является важным компонентом в поддержании и долгосрочной эффективности подобных инструментов, . Тем не менее, доступна лишь малая часть количественных данных о предполагаемых выгодах и затратах на программы по борьбе с переносчиками инфекций среди целевых групп.
Методы: Исследование проведено путем группового случайного выбора 612 домашних хозяйств в округах Гулу и Ойям в северной части Уганды в период заражения малярией, сопровождавшийся пилотной программой распыления инсектицидов остаточного действия внутри помещений (РИОДВП). В рамках исследования респонденты указывали свои предпочтения в отношении различных программ РИОДВП в зависимости от гипотетической

денежной компенсации, установленной в экспериментальном порядке. Данные были проанализированы с использованием условной модели логит-регрессии с целью оценки готовности принять компенсацию (ГПК) в денежном выражении и сократить риски распространения малярии. Модели скрытого класса использовались для анализа ответов респондентов и предполагаемого ГПК.
Результаты: Средняя ГПК оценивается в $8.94 ежегодно с 10%-ым снижением риска малярии, дополнительные компенсации РИОДВП оценивались в среднем в $54-$56 (в зависимости от типа инсектицидов) за 1 цикл РИОДВП. Наблюдается значительная неоднородность: Четыре из пяти глав домашних хозяйств в северной части Уганды имеют высокую значимость для программ IRS, в то время как остальные 20% страдают от дорогостоящих побочных эффектов РИОДВП (на сумму $2 и $3 за цикл). Статистически значимыми факторами принадлежности к группе высокой значимости являются: пол респондента, средний возраст членов семьи, участие в предыдущих РИОДВП, базовые знания в области размножения комаров и количество противомоскитных сеток в наличии. Такие факторы, как доход или благосостояние, не являются статистически значимыми предпосылками ГПК.
Выводы: Данное исследование свидетельствует о том, что для большинства людей в районах с высокими показателями заболевания малярией, таких как северная часть Уганды, программы по борьбе с переносчиками инфекций с применением РИОДВП имеют большое значение. Однако, существует значительная неоднородность с точки зрения предполагаемых побочных эффектов (положительных и отрицательных). Это может повлиять на сохранение общественной поддержки для осуществления подобных программ в долгосрочной перспективе.

Translated from English version into Russian by Daria Toropchyn, through

TRANSLATORS
WITHOUT BORDERS

Percepciones del hogar y valoraciones subjetivas de los programas de fumigación de interiores para el control de la malaria en el norte de Uganda

Zachary S. Brown, Randall A. Kramer, David Ocan, Christine Oryema

Resumen

Antecedentes: Las técnicas basadas en los insecticidas siguen siendo fundamentales para el control de las enfermedades transmitidas por vectores en Uganda. Asegurar el apoyo público de las poblaciones donde son utilizadas estas técnicas es un componente importante en el mantenimiento de su eficacia a largo plazo. Sin embargo, hay poca evidencia cuantitativa disponible sobre los beneficios percibidos y los costos de los programas de control de vectores entre los hogares seleccionados .
Métodos: Se estudió una muestra aleatoria de un grupo de 612 hogares en los distritos de Gulu y

Oyam en el norte de Uganda, durante un período de transmisión muy alto de malaria y después de un programa piloto de rociado residual de interiores (IRS). Dentro del estudio, se llevó a cabo un experimento de elección discreto en el que los encuestados indicaron sus preferencias por los diferentes programas del IRS en relación con la compensación económica, en una serie experimentalmente controlada de conjuntos de elección hipotéticos Los datos se analizaron mediante modelos de regresión logística condicional, para estimar la disposición de los encuestados a aceptar (WTA) una cierta compensación económica por reducir el riesgo de malaria. Se utilizaron modelos de clase latentes para analizar si las características de los encuestados predecían la WTA.

Resultados: La WTA promedio se estima en $ 8,94 al año, para una reducción del 10% en el riesgo de malaria, y se estimaron cobeneficios adicionales del IRS por un valor promedio de $ 54 **-** $ 56 (dependiendo del tipo de insecticida) por ronda de IRS. Se observó una heterogeneidad significativa: cuatro de cada cinco jefes de hogar en el norte de Uganda tienen altas valoraciones de los programas de IRS, mientras que el remanente 20% experimenta efectos secundarios costosos del IRS (valorado entre $ 2 y $ 3 por ronda). Predictores estadísticamente significativos para pertenecer al grupo de alta valoración incluyen, el género de los participantes, la edad media de los miembros del hogar, la participación en IRS anteriores, los conocimientos básicos sobre la reproducción de los mosquitos y el número de mosquiteros que tienen en propiedad. La representación de los ingresos del hogar y la riqueza no se encuentran dentro de los predictores estadísticamente significativos de la WTA

Conclusiones: Este estudio sugiere que la mayoría de las personas en las zonas de alta transmisión de malaria, tales como el norte de Uganda, dan un gran valor a los programas de control de vectores utilizando IRS. Sin embargo, existe una significativa heterogeneidad en términos de los efectos secundarios percibidos (positivos y negativos). Esto tiene implicaciones para mantener el apoyo público a estos programas en el largo plazo.

Translated from English version into Spanish by patriciacassoni, through

TRANSLATORS WITHOUT BORDERS
