# Supplementary material for: Household perceptions and subjective valuations of indoor residual spraying programmes to control malaria in northern Uganda
Source: Infect Dis Poverty. 2016 Oct 6;5:100. doi: 10.1186/s40249-016-0190-1 (PMC5053089; doi:10.1186/s40249-016-0190-1)
Supplement: Supplementary file 2 — Supplementary material. (PDF 341 kb) [file 40249_2016_190_MOESM2_ESM.pdf]

Supplementary Material for:

Household perceptions and subjective valuations of indoor residual spraying programs to control malaria in northern Uganda

1 Income and asset indices

The survey contained two separate estimates of household money income (questions 12 and 63), a measure of expenditure (question 64) as well as an inventory of household assets with respondents' subjective valuations of resale value (question 35). The income and expenditure data were combined into one composite income measure using principal components analysis (pca): the composite measure is a weighted average of the two income and expenditure variables, with the weights determined by the pca. Using alternative income measures in the regression analyses of the paper does not appreciably change the main regression results.

2 Random utility choice models and estimation results

Table A1 presents the raw regression coefficient estimates from the conditional logit regression model (equation 1 in the manuscript) generating the WTA results in Table 4 of the manuscript. The WTA estimates in Table 4 were obtained by dividing each attribute's coefficient reported in Table A1 by the coefficient on money, β_{money}, and then multiplying by a discount rate of 10%, using an estimate from Bauer and Chytilová (2010) according to eq. (6) in the manuscript. Standard errors for the WTA estimates are obtained using the delta method (implemented via the *nlcom* command in Stata).

3 Implementing the latent class models, sampling weights and calculating WTA

The full latent class model, with parameters consisting of β^l and γ_l for each class l (with the restriction that reference class has $\gamma_0 = \mathbf{0}$), is estimated using an expectation-maximization (EM) algorithm with respect to the above expected log-likelihood. Details can be found, for example, in: (Burton & Rigby, 2008; Morey, Thacher, & Breffle, 2006; Train, 2009). The Class 1 and 2 columns in Table 4 of the manuscript correspond to WTA estimates calculated from the preference parameters β^l, and 'marginal effect' column on the far left of Table 5 corresponds to marginal effect estimate based on γ_l, i.e. $n^{-1}\sum_{i=1}^{n}\partial\pi_{i,l}/\partial z$ for respondent covariate z. Standard errors are calculated using the delta method. The marginal effect of each covariate on annualized WTA (the last three columns of Table 5) are calculated as $\frac{\delta}{n}\sum_{i=1}^{n}\sum_{l}\left(\frac{\partial\pi_{i,l}}{\partial z}\right)\frac{\beta_x^l}{\beta_{money}^l}$, where β_x^l is the preference coefficient for class l members with respect to attribute x (Scarpa, Willis, & Acutt, 2005).

Sampling weights cannot be directly applied with the latent class statistical package used (the *lclogit* package written for Stata®). However, in the latent class model, the sampling weights should affect the distribution of respondents between the classes more than the preference parameters within each class. Thus sampling weights were used postestimation by taking predicted posterior class membership probabilities $\hat{\rho}_{i,l}$ from the latent class model and regressing these again on z_i using a multinomial logit model (using the *fmlogit* package in Stata), but now with sampling weights applied (using the *pweight* option in Stata). The results with and without sampling weights are very similar in terms of coefficient magnitudes and statistical significance.

4 Institutional review board for human subjects research

This research was approved by the Duke University Institutional Review Board (under protocol #2699). Survey respondents gave their informed consent to participate in the study (see informed consent statement in the survey questionnaire, attached as a supplement). All individually identifying information was destroyed at the end of the study.

References

Bauer, M., & Chytilová, J. (2010). The impact of education on subjective discount rate in ugandan villages. *Economic Development and Cultural Change*, *58*(4), 643–669.

Burton, M., & Rigby, D. (2008). Hurdle and Latent Class Approaches to Serial Non-Participation in Choice Models. *Environmental and Resource Economics*, *42*(2), 211–226. http://doi.org/10.1007/s10640-008-9225-9

Morey, E., Thacher, J., & Breffle, W. (2006). Using Angler Characteristics and Attitudinal Data to Identify Environmental Preference Classes: A Latent-Class Model. *Environmental and Resource Economics*, *34*(1), 91–115. http://doi.org/10.1007/s10640-005-3794-7

Scarpa, R., Willis, K. G., & Acutt, M. (2005). Individual-specific welfare measures for public goods: a latent class approach to residential customers of Yorkshire Water. *Econometrics Informing Natural Resource Management', Edward Elgar Publisher, Cheltenham, UK and Nottingham, MA, USA*.

Train, K. E. (2009). *Discrete Choice Methods with Simulation*. Cambridge University Press.

Table A1. Random utility choice model estimation results.

	Conditional logit model[1]		*Latent class logit model*[2]			
			Class 1 (High WTA)		Class 2 (Low WTA)	
Alternative-specific attributes	Coeff.	Std. Err.	Coeff.	Std. Err.	Coeff.	Std. Err.
Malaria risk	-0.744***	(0.199)	-1.638***	(0.304)	-0.416	(0.336)
Compensation (USD)	0.000832***	(0.000208)	0.000847***	(0.000202)	0.0108***	(0.00201)
Rounds of IRS per year						
DDT-based	0.469***	(0.0402)	0.741***	(0.0416)	-0.214***	(0.0825)
ICON-based	0.447***	(0.0319)	0.714***	(0.0409)	-0.303***	(0.0915)
Predicted class sizes						
Unconditional			80%		20%	
Unconditional (survey weights)			82%		18%	
Conditional			81%		19%	
Conditional (survey weights)			84%		16%	
Respondents	588		588			
Choice tasks per respondent	3		3			
Model degrees of freedom	4		25			
Log-likelihood	-1,376		-1,166			

Notes: ***, ** and * indicate statistical significance at the 1%, 5% and 10% levels, respectively. Standard errors calculated clustering at the respondent level (i.e. across choice tasks). [1]Model estimated with sampling weights. Model estimated without sampling weights yields similar results, but with a 34% lower (in magnitude) malaria risk coefficient and a log-likelihood value of -1,419. [2] Model and reported log-likelihood first estimated without sampling weights, due to software limitations. To account for sampling design, sampling weights applied to class membership model (see Table 5 in manuscript).
