# Supplementary material for: Household perceptions and subjective valuations of indoor residual spraying programmes to control malaria in northern Uganda
Source: Infect Dis Poverty. 2016 Oct 6;5:100. doi: 10.1186/s40249-016-0190-1 (PMC5053089; doi:10.1186/s40249-016-0190-1)
Supplement: Supplementary file 3 — Understanding households’ preferences regarding indoor residual spraying, malaria-related risks, and other risks. (PDF 1056 kb) [file 40249_2016_190_MOESM3_ESM.pdf]

Understanding households' preferences regarding indoor residual spraying, malaria-related risks, and other risks
(to be administered orally by enumerators to respondents)

October 2009

Introduction

Hi, I am _____. I am a member of a research team from a university in the United States and Gulu University. We know that malaria is a problem in this community. We also know that you have information and ideas that could help us think about how to solve the problem and hope that you will be willing to talk with us. If you agree, we think we would need about an hour of your time. We would like you to participate in a discussion about a number of different topics that we think might help us understand the problem. Some of the questions are personal questions, such as whether any members of your household have had malaria and your primary source of family income, some questions are about your opinions, for example, your thoughts about government programs to stop malaria. The results from this survey will ultimately help to design programs which are more effective and more accepted by the people here.

We are also interested in the way land is used by households in this area, because this affects the ability of that land to provide for the people here and because land use decisions can affect the risk of malaria facing the people here.

If you decide to participate in this survey, we will need to record the location of your household on a map. This information will be secured so that only we, the researchers, will have the keys to a locked file cabinet and an encrypted computer file where the survey results, including your household's location, will be stored. If you choose not to participate in this survey, that is fine too. If you participate, you are free to skip any questions you do not wish to answer or to stop any time.

GG Do you wish to participate? ☐ ☐ c(108)

Yes *No*

If "Yes," proceed with survey. If "No," thank the respondent and go to the next household.

<u>TO BE COMPLETED BEFORE BEGINNING INTERVIEW</u>

Questionnaire # ☐☐☐ **Survey Version** *0 1*

Date___/___//___/___//____/___/___/___// DD/MM/YYYY c(109/16)

Name of the interviewer ______________________________________

District ______________________**(117,19)** **Sub-county** ______________________**(120,22)**

Parish __________________**(123,25)** **Village** ______________________**(126.29)**

GPS Coordinates:

N				*o*			.				'	*(131,40)*
E				*o*			.				'	*(141,50)*

<u>Time at start</u>:__________**HH/MM c(151/54)** **<u>Time at finish</u>:**________**HH/MM c(155/58)**

I. Demographics and Education

1. *Household roster, structure, education, and labor, and malaria history. Enumerator reads the following script:*

   "We are first going to ask you some questions about who is in your household. We first want to know who normally shares cooked meals with you, how old these people are, and how they are related to you. For each of these people, we would like to know some general information on how much school they have attended, whether they have had malaria recently, and what they do with their time."

   [Continue to next page.]

For each person who normally shares a cooked meal with the respondent, please complete the following:

House hold Member #	1.1 Age (years)<br>95 = "Don't know"	1.2 Sex<br>(1) Male<br>(2) Female	1.3 Relation to head of HH:<br>(1) Head<br>(2) Spouse<br>(3) Brother / sister<br>(4) Parent<br>(5) Biological child, or grandchild<br>(6) Others' child in the care of the respondent<br>[95] Other<br>[98] No response	1.4 Marital Status<br>(1) Married<br>(2) Single<br>(3) Cohabitating<br>(4) Separated<br>(5) Divorced<br>(6) Widowed<br>[95] Other<br>[98] No response<br>[00] Not applicable	1.5 & 1.6. Highest grade attended and completed?<br>*Circle highest grade attended. Check column 1f.only if completed.*<br>(0) Never attended school, *open circle only*<br>(1) Primary (1-7)<br>(2) Secondary (1-4)<br>(3) High School (1-2)<br>(4) Technical education<br>(5) Undergraduate study /university (1-8)<br>(6) Graduate study<br>[99] Don't know<br>[98] No response		1.7 Did this person suffer from malaria in the past month?<br>(1) No<br>(2) Yes, went to the health facility where they took blood.<br>(3) Yes, diagnosed by health worker at the health facility, but no blood taken.<br>(4) Yes, diagnosed by traditional healer<br>(5) Yes, friend or relative diagnosed it<br>(6) Yes, we diagnosed it ourselves based on the symptoms<br>[99] Don't know<br>[98] No response	CARDS
					1.5 Highest Attended	1.6 Completed?<br>[1] Yes [2] No		
1*		1 2	1 2 3 4 5 6 95 98	1 2 3 4 5 6 95 98 00	0 1 2 3 4 5 6 99 98	1 2	1 2 3 4 5 6 99 98	(3)
2		1 2	1 2 3 4 5 6 95 98	1 2 3 4 5 6 95 98 00	0 1 2 3 4 5 6 99 98	1 2	1 2 3 4 5 6 99 98	(4)
3		1 2	1 2 3 4 5 6 95 98	1 2 3 4 5 6 95 98 00	0 1 2 3 4 5 6 99 98	1 2	1 2 3 4 5 6 99 98	(5)
4		1 2	1 2 3 4 5 6 95 98	1 2 3 4 5 6 95 98 00	0 1 2 3 4 5 6 99 98	1 2	1 2 3 4 5 6 99 98	(6)
5		1 2	1 2 3 4 5 6 95 98	1 2 3 4 5 6 95 98 00	0 1 2 3 4 5 6 99 98	1 2	1 2 3 4 5 6 99 98	(7)
6		1 2	1 2 3 4 5 6 95 98	1 2 3 4 5 6 95 98 00	0 1 2 3 4 5 6 99 98	1 2	1 2 3 4 5 6 99 98	(8)
7		1 2	1 2 3 4 5 6 95 98	1 2 3 4 5 6 95 98 00	0 1 2 3 4 5 6 99 98	1 2	1 2 3 4 5 6 99 98	(9)
8		1 2	1 2 3 4 5 6 95 98	1 2 3 4 5 6 95 98 00	0 1 2 3 4 5 6 99 98	1 2	1 2 3 4 5 6 99 98	(10)
9		1 2	1 2 3 4 5 6 95 98	1 2 3 4 5 6 95 98 00	0 1 2 3 4 5 6 99 98	1 2	1 2 3 4 5 6 99 98	(11)
10		1 2	1 2 3 4 5 6 95 98	1 2 3 4 5 6 95 98 00	0 1 2 3 4 5 6 99 98	1 2	1 2 3 4 5 6 99 98	(12)
11		1 2	1 2 3 4 5 6 95 98	1 2 3 4 5 6 95 98 00	0 1 2 3 4 5 6 99 98	1 2	1 2 3 4 5 6 99 98	(13)
12		1 2	1 2 3 4 5 6 95 98	1 2 3 4 5 6 95 98 00	0 1 2 3 4 5 6 99 98	1 2	1 2 3 4 5 6 99 98	(14)
13		1 2	1 2 3 4 5 6 95 98	1 2 3 4 5 6 95 98 00	0 1 2 3 4 5 6 99 98	1 2	1 2 3 4 5 6 99 98	(15)
14		1 2	1 2 3 4 5 6 95 98	1 2 3 4 5 6 95 98 00	0 1 2 3 4 5 6 99 98	1 2	1 2 3 4 5 6 99 98	(16)
15		1 2	1 2 3 4 5 6 95 98	1 2 3 4 5 6 95 98 00	0 1 2 3 4 5 6 99 98	1 2	1 2 3 4 5 6 99 98	(17)
**c(11/12)**	**c(13/14)**	**c(15)**	**c(16/17)**	**c(18/19)**	**c(20/21)**	**c(22)**	**c(23/24)**	

* Respondent

2. Please list any special skills or training, which anybody in the household possesses.
   (Do not read. Circle all that apply.)
   c(2108/ 39)
   [01] Bicycle repair
   [02] Motorcycle / car repair
   [03] Carpentry
   [04] Sewing
   [05] Animal raising
   [06] Agricultural training
   [07] Electrical
   [08] Building
   [09] Mechanical
   [10] Nursing
   [11] Trained birth attendant
   [12] Hunting
   [13] Other
   [99] Don't know
   [98] No response

3. Religion?
   c(2140, 41)
   [1] Christian
   [2] Muslim
   [3] No religion / pagan
   [98] No response

4. What tribe does this household belong to? ______________________________ c(2142/44)

5. Are any household members from other tribes? If so, which ones?

   ____ c(2145/56)

II. General Assessment of Subjective Expectations and Income

Enumerator script:

"I am going to ask you several questions about the chance or likelihood that certain events are going to happen. There are 30 ground nuts in the cup. For a given event, I would like you to choose some ground nuts out of these 30 and put them in the plate to express what you think the likelihood or chance is of a specific event happening. One bean represents one chance out of 30. If you do not put any ground nuts in the plate, it means you are completely sure that the event will NOT happen. As you add ground nuts, it means that you think the likelihood of the event increases. If there are more ground nuts in the cup than on the plate, then it means you think the event is not likely to happen. If there are more ground nuts on the plate than in the cup, then it means you think the event is likely to happen. If you put all of the ground nuts on the plate, then it means you think it is IMPOSSIBLE that the event will NOT happen. There is no right or wrong answer. I just want to know what you think."

6. How likely is it that it will rain tomorrow?

   Number of ground nuts: ________ of 10 c(2208/09)

7. How likely is it that it will rain at least once within the next week, **INCLUDING TOMORROW**?

   Number of ground nuts: ________ of 10 c(2210/11)

8. How likely is it that it will rain at least once during the weeks before or after March 15th?

   Number of ground nuts: ________ of 10 c(2212/13)

9. How likely is it that it will rain at least once during the weeks before or after on July 15th?

   Number of ground nuts: ________ of 10 c(2214/15)

Result of Coin Flip | *Heads* | 1 | *Tails* | 2 *c*(2221)

IF THE COIN FLIP COMES UP HEADS, PLEASE PROCEED TO QUESTION 10 FIRST AND THEN QUESTION 11, OTHERWISE ASK QUESTION 11 FIRST FOLLOWED BY 10:

10. *[PLACE 8 RED CHIPS AND 2 WHITE CHIPS IN ONE GROUP ON THE TABLE/GROUND IN FRONT OF THE ENUMERATOR. IN FRONT OF THE RESPONDENT, PLACE A PILE OF 10 "TREATS"(E.G. SOAPS, CANDIES, ETC.). WAIT UNTIL EVERYTHING IS READY BEFORE CONTINUING...]* As you can see, there is \a group of chips in front of me with 8 red chips and 2 white chips. I want you to divide up your pile of treats into two piles, one on your left and one on your right. Then I want you to close your eyes and draw a chip from the bag. If you draw a red chip from the bag, you will get to keep the pile on your left. If I draw a white chip, you will get to keep the one on your right. There is no right or wrong answer; we just want to know which group of chips you prefer us to put in the bag.

Respondent's Left Pile / **RED CHIP WINS** | **Respondent's Right** Pile / **WHITE CHIP WINS**

[ ] | [ ] c(2222)

IF COIN FLIP CAME UP HEADS, READ THE FOLLOWING TO THE RESPONDENT, BEFORE CONTINUING TO QUESTION 11: We are now going to play the same game as before, except that you can now see that the pile of chips in front of me is different:

11. *[CLEAR THE SPACE IN FRONT OF YOU AND THE RESPONDENT. THEN PLACE 4 RED CHIPS, 1 WHITE CHIP, AND 5 GREEN CHIPS IN ONE GROUP ON THE TABLE/GROUND IN FRONT OF YOU. IN FRONT OF THE RESPONDENT, PLACE A PILE OF 10 TREATS. WAIT UNTIL EVERYTHING IS READY BEFORE CONTINUING...]* There are now 4 red chips, 1 white chips, and 5 green chips. Just as before, I want you to divide up your pile of treats into two piles, one on your left and one on your right. If I draw a red chip from the bag, you will get to keep the pile on your left. If I draw a white chip, you will get to keep the one on your right. However, you can see that there are green chips in the pile in front of me. If I draw a green chip from the bag, I will take away both of your piles. Go ahead divide up your piles.

Respondent's Left Pile / **RED CHIP WINS** | **Respondent's Right** Pile / **WHITE CHIP WINS**

[ ] | [ ] c(2223)

IF COIN FLIP CAME UP TAILS, PROCEED TO QUESTION 10 AFTER READING THE FOLLOWING TO THE RESPONDENT: We are now going to play the same game as before, except that you can now see that the pile of chips in front of me is different:

[AFTER COMPLETING QUESTIONS 10 AND 1, REMOVE COLORED CHIPS AND TREATS, AND REPLACE GROUND NUTS.]

12. What is the highest and lowest amount in Ugandan shillings you might receive as compensation for wage labor, goods you will sell, or as a gift **<u>IN AN AVERAGE MONTH</u>** during the rainy season? During the dry season?

<u>FOR THIS QUESTION AND FOLLOWING, DEFINE THE SEASONS AS FOLLOWS:</u>

DRY SEASON **NOVEMBER 15 – MARCH 15**
WET SEASON **MARCH 15 – NOVEMBER 15**

Amounts in Ugandan shillings:

	**Wet** season								**Dry** Season						
Highest Amount (UGX)								c(2231/37)							c(2251/57)
Lowest Amount (UGX)								c(2241/47)							c(2261/67)

[Enumerator computes simple average of each column using calculator]:

c(2311/17) c(2321/27)

	**Wet** season							**Dry** Season						
Averages of above table														

13. Using the ground nuts in front of you, indicate the likelihood that you will earn or receive at least *[averages computed in question 12]* Ugandan shillings worth of goods and services over an average month in the wet season? In the dry season?

c(2331/ 37*)* c(2341/ 47*)*

	**Wet** season							**Dry** Season						
Number of ground nuts out of 10														

III. Malaria Knowledge, Experience, Subjective Risk Assessment, and Treatment

Malaria Knowledge

14. What do you think causes malaria? *(DO NOT READ ANSWERS, CIRCLE ALL THAT APPLY.)*

c(2408/29)
[01] Mosquitoes
[02] Bad water
[03] Evil Spirits
[0 4] Playing in the rain
[05] Flea/Tick bite
[06] Unclean environment
[07] Cockroaches
[08] Eating mangoes
[09] Too much time in the rain
[10] Cold weather or food
[95] Other_______________
[99] Don't know

15. Are mosquitoes present in your household or community?

c(2430)
[1] Yes
[2] No *(GO TO QUESTION 17.)*
[9] Don't know

16. What factors do you think affect the mosquitoes in your community and around your home? *(DO NOT READ ANSWERS, CIRCLE ALL THAT APPLY.)*

c(2431/49)
[01] Amount of rainfall
[02] Amount of standing/still water
[03] Farming methods
[04] Cleanliness of village or household surroundings/dirty environment
[05] Temperature
[06] Uncleared brush, grass
[95] Other_____________
[99] Don't know

Malaria Subjective Risk Assessment and Spraying

17. Some households in this area have been visited by government teams which spray homes with insecticides to eliminate the mosquitoes which cause malaria. Did you or someone else ever allow a team to spray your home?
    c(2450)
    [1] Yes
    [2] No
    [9] Don't know

18. Do you think that spraying your home with insecticides could help reduce the chance that you or some member of your household would get malaria?
    c(2451)
    [1] Yes
    [2] No
    [9] Don't know

19. Please indicate what you think the likelihood is that a person in your household will get malaria at any point in the **NEXT MONTH** if your home **IS NOT** sprayed by one of the government teams; the number of ground nuts that you put in the plate means that you think that many people out of 10 will get malaria.

    Number of ground nuts **remaining on the plate**: ________ of 10 c(2452/53)

IF ANSWER TO QUESTION 18 WAS [2], SKIP TO NEXT PAGE.

20. Please indicate what you think the likelihood is that a person in your household will get malaria at any point in the **NEXT MONTH** if your home **IS** sprayed by one of the government teams; the number of ground nuts that you put in the plate means that you think that many people out of 10 will get malaria.

    Number of ground nuts **remaining on the plate**: ________ of 10 c(2454/55)

IV. IRS Choice experiments

EXPLAIN THE FOLLOWING TO THE RESPONDENT:

Requirements to participate in the spray program:

- Have 10 litres of water to provide to the spray team when they arrive. They would come in the morning or afternoon, but you would be warned by radio and the LC1 in advance.
- Households must remove all of their belongings from their home. This includes all food, garments, beds, cooking tools, chairs, and toothbrushes.
- Households must remain outside of their homes for 2 hours while the teams do their work.
- Households must not re-cover their floors and walls with dung or other stuff. Doing so eliminates the protective effect of the insecticide.

Properties of DDT (*dudumaki*):

- Was used for many years in Africa and other places in agriculture.
- May hurt people's health over a long time (e.g. *cancer*), but scientists aren't sure.
- Approved for use in spraying homes by the Ugandan government.
- Can harm/kill animals, such as birds.
- Can kill many other insects in addition to mosquitoes that cause malaria, such as cockroaches and bedbugs.
- Its effects—good and bad—last for a very long time.

Properties of ICON:

- Its effects—good and bad—last for a shorter time than DDT.
- May hurt people over a long time, but this effect is not as bad as DDT, because the chemical lasts for a shorter time.
- Approved for use in spraying homes by the Ugandan government.
- Can harm/kill animals, such as fish.
- Only kills the mosquitoes which cause malaria, not other insects.
- Crops with high amounts of DDT may get a lower price if sold or "exported" to other parts of the world.

<u>COUNT OUT 30 GROUND NUTS (20 MORE THAN THE 10 THAT ARE ALREADY OUT). DIVIDE THE PILE OF 30 GROUND NUTS UP INTO THREE PILES OF 10 ON THE MAT.</u>

"We want to ask you questions about things the government could do to help prevent malaria."

21. ***<u>FOR EACH OF THE THREE CHOICE TASKS IN THE TABLE BELOW, READ THE FOLLOWING TO THE RESPONDENT:</u>***

First suppose that you were offered three options:

<u>In the first option</u>, your house would be sprayed with **the chemical *[first row of the choice task, column i]*** in such a way that the chance of getting malaria in the next month would be ***[first row of the choice task, column iii]***. In order to maintain the malaria-prevention effect of the insecticide, the spray teams would have to return every ***[first row of the choice task, column ii]*** in order to spray your home again.

<u>In the second option</u>, your house would also be sprayed but with ***[second row of the choice task, column i]*** in such a way that the chance of getting malaria in the next month was ***[second row of the choice task, column iii]***. In order to maintain the malaria-prevention effect of the insecticide, the spray teams would have to return every ***[second row of the choice task, column ii]*** in order to spray your home again.

<u>In the third option, you would be given a one-time payment of *[third row of the choice task, column iv]* Ugandan schillings to use for anything you liked, including things to prevent malaria (sprays, bednets, etc.). Since there would be no insecticide spraying in this case, the risk of malaria would be higher, with *[third row of the choice task, column iii]*.</u>

<u>FOR EACH OPTION, THE NUMBER OF CHIPS REPRESENTS THE NUMBER OF RE-SPRAYING REQUIRED FOR THAT OPTION. FOR THE MONEY OPTION, USE A GREEN CHIP.</u>

<u>COLOR CODES FOR THE CHIPS:</u>

WHITE = DDT
RED = ICON
GREEN = MONEY

	<u>CIRCLE ONE NUMBER FOR EACH TASK</u>	*i. Insecticide*	*ii. Number of Re-sprayings per year*	*iii. Malaria risk <u>PER MONTH</u>*	*iv. Compensation*	*CARD*
<u>TASK A</u>	*1*	*DDT*	*1*	*8 people sick out of 10*	*0*	*(26)*
	2	*DDT*	*4*	*7 people sick out of 10*	*0*	*(27)*
	3	*--*	*0*	*9 people sick out of 10*	*10,000 UGX*	*(28)*
	-10	*Would not participate*				
<u>TASK B</u>	*1*	*--*	*0*	*9 people sick out of 10*	*100,000 UGX*	*(29)*
	2	*DDT*	*4*	*5 people sick out of 10*	*0*	*(30)*
	3	*DDT*	*1*	*6 people sick out of 10*	*0*	*(31)*
	-10	*Would not participate*				
<u>TASK C</u>	*1*	*DDT*	*1*	*3 people sick out of 10*	*0*	*(32)*
	2	*--*	*0*	*8 people sick out of 10*	*100,000 UGX*	*(33)*
	3	*ICON*	*4*	*2 people sick out of 10*	*0*	*(34)*
	-10	*Would not participate*				
	c(11/12)	*c(13/14)*	*c(15)*	*c(16/17)*	*c(21/27)*	

22. If you were to get medication for a household member's malaria in the future, where would you get this medication? ***(DO NOT READ ANSWERS)***

c(3508/19)

[01] Wouldn't use any medication ***(GO TO QUESTION 25.)***
[02] Health facility (examples: SOS)
[03] Traditional healer
[04] Drug store
[05] Used local herbs gathered by members in the household
[06] Leftovers from previous sick person, or from others, such as friends or neighbors

23. How much do you expect it would cost you to obtain enough of this treatment to help you until your symptoms went away?

Ugandan shilling: [| | | | | | |] c(3521/27)

IF ANSWER TO QUESTION 22 WAS [2], [4], OR [6], ASK QUESTION 24. OTHERWISE, SKIP TO QUESTION 25.

24. Do you know the name for this type of medication?
(DO NOT READ ANSWERS, CIRCLE ANY THAT APPLY.)

C(3530/39)

[01] Coartem
[02] Quinine
[03] Chloroquine
[04] Other
[99] Don't know

25. Who in your household uses a mosquito net?
(DO NOT READ ANSWERS, CIRCLE ALL THAT APPLY)

C(3540/59)

[01] Children under 5
[02] Pregnant women
[03] Children and women
[04] Men
[05] Everyone
[06] No one *(GO TO QUESTION 28)*
[07] Other ____________
[99] Don't know

26. How frequently do you (your family) sleep under these nets?
(*READ ANSWERS, CIRCLE ONLY ONE)*

c(3560)

[1] Every night
[2] Most nights (4 or more nights per week)
[3] Some nights (3 or less nights per week)
[9] I don't know

27. Around what time do you (your family) go under the nets (go to bed)?

Time (in hours): ___________ HH/MM c(3561/64)

V. Wealth and assets other than land

28. If you needed 200,000 UGS, would you or your household be able to raise it using any of the following means? *[READ ALL OPTIONS.]*

	Codes:<br>(1) Yes<br>(2) No<br>[99] Don't know<br>[98] No response	
Borrow from family	1 2 99 98	c(3608/09)
Borrow from friends	1 2 99 98	c(3610/11)
Borrow from bank	1 2 99 98	c(3612/13)
Sales of valuables owned by the household	1 2 99 98	c(3614/15)

29. Floor material: *(OBSERVE, DON'T ASK)*

c(3621/22)

(01) Natural floor (examples: earth, sand, dung)
(02) Rudimentary floor (examples: wood planks, palm, bamboo)
(03) Finished floor (parquet or polished wood, vinyl or asphalt strips, ceramic, cement, carpet)
[95] Other_______________

30. Size of house: *(OBSERVE, DON'T ASK)*

c(3623)

(1) Large (4 or more rooms)
(2) Medium (2 or 3 rooms)
(3) Small (1 room)

31. Type of wall: *(OBSERVE, DON'T ASK)*

c(3624/25)

(01) Thatch
(02) Wood
(03) Mud or mud bricks
(04) Burnt bricks
(05) Concrete bricks
[95] Other_________________

32. Type of window: *(OBSERVE, DON'T ASK)*
c(3626/27)
(01) No windows
(02) Uncovered windows (window but no shutters)
(03) Window with wooden shutter
(04) Window with glass shutter
(05) Window with mosquito screen
(06) Window with mosquito screen and shutters
[95] Other

33. Type of roof: *(OBSERVE, DON'T ASK)*
c(3628/29)
(01) Thatch
(02) Tile
(03) Corrugated iron
(04) Tin
[95] Other_________________

34. What does your household use as its main source of fuel for cooking?
(*DO NOT READ ANSWERS, CIRCLE ONLY ONE*)
c(3630/31)
(01) Electricity
(02) Natural Gas
(03) Biogas
(04) Kerosene
(05) Coal
(06) Charcoal
(07) Firewood/straw
(08) Dung
[95] Other_______________

35. *[Assets]* I am going to read a list of items. For each item, please tell me whether anybody in your household **PRESENTLY** owns at least one of these items, and—if anybody owns the item—how much money you think could be raised by selling it.

Q#	ITEM		# Owned	Resale Value per each (in UGS)	Q#	ITEM		# Owned	Resale Value per each (in UGS)	CARD
		**c(8)**	**c(9/10)**	**c(11/12)**			**c28)**	**c(29/30)**	**c(31/40)**	
34.1	Mosquito net (*Tankweyo*)	1			34.2	Cell phone	1			c(38)
34.3	Radio / cassette player	2			34.4	Plough	2			c(39)
34.5	Bed	3			34.6	Jerry can	3			c40)
34.7	Blankets	4			34.8	Motorbike	4			c(41)
34.9	Sewing machine	5			34.10	Chair	5			c(42)
34.11	Hoe	6			34.12	Cupboard	6			c(43)
34.13	Slingshot (*Labutida*)	7			34.14	Panga / machete	7			c(44)
34.15	Bicycle	8			34.16	Rifles (*Muduku*)	8			c(45)
34.17	Tables	9			34.18	Torch / flashlight	9			c(46)
34.19	Mattress	1			34.20	Tractor	1			c(47)
34.21	Bow and arrows	2			34.22	Trap	2			c(48)
34.23	Knife	3			34.24	Wheelbarrow	3			c(49)

VI. Subsistence Questions

36. ***ADD 14 GROUND NUTS TO THE 10 ON THE MAT, SO THAT NOW THERE ARE 24 GROUND NUTS. FLIP OVER THE MAT SO THAT COMPARTMENTS FOR THE VARIOUS ACTIVTIES ARE VISIBLE.***

READ TO RESPONDENT: "Divide the ground nuts in the plate to indicate how your household allocates time for the following activities in a typical day. Each ground nut represents one hour. A higher number of ground nuts for an activity would indicate that your household spends more time doing that activity, whereas a lower number would indicate that you don't spend that much time on that activity."

CHECK WHETHER THE TOTAL NUMBER OF GROUND NUTS EQUALS 24 PER ROW

	DRY SEASON (NOVEMBER 15 – MARCH 15)					WET SEASON (MARCH 15 – NOVEMBER 15)					
ACTIVITY:	Crops and live-stock c(11/12)	Cooking and cleaning around home c(13/14)	Fuelwood collection c(15/16)	Leisure & Sleep c(17/18)	Other c(19/20)	Crops and live-stock c(31/32)	Cooking and cleaning around home c(33/34)	Fuelwood collection c(35/36)	Leisure & Sleep c(37/38)	Other c(39/40)	CARD
Primary income earner											(51)
Other co-resident adults (14 years and older)											(52)
Children (above 5 years of age and younger than 14)											(53)

37. Does anyone in your household work for wages? c(5408)

[1] Yes >>>>>>>>>***IF YES COMPLETE THE FOLLOWING:***

[2] No

	**DAYS PER WEEK DRY SEASON (NOVEMBER 15 – MARCH 15)**		**DAYS PER WEEK WET SEASON (MARCH 15 – NOVEMBER 15)**	
Primary income earner		(5411)		(5421)
Other co-resident adults (14 years and older)		(5412)		(5422)
Children (above 5 years of age and younger than 14)		(5413)		(5423)

38. Does your household hire non-household members to work for the household? c(5430)

[1] Yes, how many hours on average per day? _____________ c(5431/32)

[2] No, does not hire non-household members.

Charcoal

39. Does your household burn/make charcoal?
    c5508
    [1] Yes
    [2] No ***IF "NO," GO TO "FUELWOOD SECTION" BELOW.***
40. How many days in a month does someone in your household make charcoal, during the wet season? During the dry season?

    Dry Season: _____________ days (5509,10) Wet Season: _____________ days (5511,12)
41. During a day in which somebody in the household makes charcoal, how many hours do they actually spend on this activity?

    _______ hours per day (5513,14)
42. How do you get from your house to the most distant area where your household produces charcoal? Circle all that apply
    [01] By motorcycle [02] By bike [03] On foot [95] Other ___________ [98] No response c(5515/20)

*PICK **ONE** MODE OF TRANSPORTATION CIRCLED ABOVE AND ASK FOR THAT ONE ONLY:*

43. How long does it take you to go from your house to the most distant field where your household produces charcoal by ___________c (5521/22) [Insert code for mode of transportation from above] ?
    ______/_____ (hours) HH/MM c(5523/26)

44. How much charcoal does your family produce during a typical week?
    Dry Season:______________(basins / straw sacks *circle one*) Wet Season:_____________________ (basins / straw sacks *circle one*)

Dry Season				Wet Season			
			Basin c(5527/29)				Basin c(5534/36)
			Sacks c (5530/32)				Sacks c(5537/39)

Fuelwood APPLICABLE? [1] YES [2] NOT APPLICABLE (5540)

IF THE HOUSEHOLD INDICATED THEY SPEND TIME COLLECTING FUELWOOD, PROCEED WITH THE REMANINDER OF THIS PAG. OTHERWISE, SKIP TO NEXT PAGE.

45. How do you get from your house to the most distant area where your household collects fuelwood? *Circle all that apply*

    [01] By motorcycle [02] By bike [03] On foot [95] Other ___________ [98] No response c(5541,48)

*PICK **ONE** MODE OF TRANSPORTATION CIRCLED ABOVE AND ASK THE FOLLOWING FOR THAT ONE ONLY:*

46. How long does it take you to go from your house to the most distant field where your household collects fuelwood by ___________ (5549/50)[Insert code for mode of transportation from above] ?
    ______/_____(hours) HH/MM (5551/54)

47. How much fuelwood does your household collect during a typical week?
Dry Season: [| | |] (bundles) c(5561/64) Wet Season: [| | |] (bundles) c(5565/68)

Crops APPLICABLE? [1] YES [2] NOT APPLICABLE (5569)

IF THE HOUSEHOLD INDICATED THEY SPEND TIME GROWING CROPS, PROCEED WITH THIS PAGE. OTHERWISE, SKIP TO NEXT PAGE.

48. How large is the total land your household is using to produce crops during the current wet season?

Area (current wet season): [| | |] acres c(5611/14)

49. How do you get from your house to the most distant area where your household grows crops? Circle all that apply

[01] By motorcycle [02] By bike [03] On foot [95] Other ___________ [98] No response c(5615/24)

*PICK **ONE** MODE OF TRANSPORTATION CIRCLED ABOVE AND ASK FOR THAT ONE ONLY:*

50. How long does it take you to go from your house to the most distant field where your household grows crops by ___________ c(5625/26)[Insert code for mode of transportation from above] ?

[| | |] (min) c(5631/34)

51. Do you plan to try planting any new crops in the next planting season:
[1] Yes c(5640)
[2] No, why not: ________________ c(5641/65)

52. Do you have plans to plant any of the following crops in future years? *(LIST THE FOLLOWING. CIRCLE ALL THAT APPLY)*

C(5666/79)
[01] Pineapple
[02] Water melons
[03] Pawpaw
[04] Onion
[05] Tomato
[06] Banana

Livestock

53. Does your household have livestock?

[1] Yes [0] No *IF "NO," GO TO QUESTION 49, ON NEXT PAGE.* *c*(5708)

54. How do you feed your animals? c(5709, 20)

(01) Tethering
(02) Zero-grazing
(03) Open grazing
(04) Private grazing
[95] Other_________________

55. How many animals does your household have?

Livestock	Total #			
Cattle				c(5721/23)
Goats				c(5725/27)
Pigs				c(5729/31)
Chickens				c(5733/35)

VII Land Access and plans for the future

56. On whose land does your household perform the following activities? (<u>*Please circle all that apply.*</u>)

Activity	**Land ownership type**<br>(001) The land belongs to my household *("certified freehold land")*<br>(002) The land belongs to my parents or clan<br>(003) My household rents the land<br>(004) The land is common *("Nobody's land")*<br>(005) The land belongs to the government<br>[095] Other<br>[098] No response<br>[099] Don't know<br>[000] Not applicable	
Collect fuelwood	001 002 003 004 005 006 095 098 099 000	c(5811/34)
Cut trees for charcoal	001 002 003 004 005 006 095 098 099 000	c(5835/61)
Grow crops	001 002 003 004 005 006 095 098 099 000	c(5911/34)
Graze livestock	001 002 003 004 005 006 095 098 099 000	c(5935/61)
Hunting	001 002 003 004 005 006 095 098 099 000	c(6011/34)

57. How likely is that anybody from your household will get involved in a conflict over land in the next two weeks? *Conflict in this case is assumed to mean any form of disagreement ranging from disputes to court trials*
Number of ground nuts: ___________of 10 c(6108/ 09)

58. Who are you likely to have a conflict with over land? *DO NOT READ RESPONSES. CIRCLE ALL THAT APPLY*
C(6110,29)
[01] Other co-wives of husband
[02] Siblings
[03] Parents
[04] Children
[05] Neighbours
[06] Clan members
[07] People from another village
[95] Other_________________
[00] Not applicable
[98] No response

59. How much land does your household have documents proving ownership for?

Total area of freehold or leased land: [ ][ ][ ][ ] acres c(6131/34)

60. How likely is that another household will start using any of your land without your permission next month?

Number of ground nuts: ___________of 10 c(6140/41)

61. What is growing on your land? *(DO NOT READ. CIRCLE ALL THAT APPLY.)*

c (6208/25)

[01] Crops, other than banana
[02] Rice
[03] Native forest
[04] Forest plantation (Pine and Eucalyptus trees), other than banana
[05] Banana
[95] Other
[99] Don't know
[98] No response

62. Does your household intend to live in the same village next year? c(6230)

[1] Yes [0] No

Socio-Econ Questions

63. Please list how much income your household receives on average per season from the following activities
Because households may exchange goods or receive payments in kind rather than monetary income, please record everything a household receives as a payment in ***local units***

**Source of Income**	**Income (UGX per (Dry Season) (March 15- November 15)**		**Income (UGX per Wet Season) (March 15- November 15)**		
	**Min**	**Max**	**Min**	**Max**	**CARD**
Sale of fuelwood					**(64)**
Sale of charcoal					**(65)**
Sale of construction materials (poles, grass roofs and bricks)					**(66)**
Sale of crops					**(67)**
Sale of livestock					**(68)**
Petty business					**(69)**
Other non-wage income					**(70)**
Wage Earnings					**(71)**
	**c(11/20)**	**c(21/30)**	**c(31/40)**	**c(41/50)**	

64. Please list the minimum and maximum expenses your household incurs per season

Because households may pay in kind rather than with money, please record everything a household pays in local units

Please list the average expenses your household incurs **per season**			
	**Min**	**Max**	
Education fees	c(7311/20)	c(7321/30)	Per term
Clothes	c(7331/40)	c(7341/30)	Per year
Shelter	c(7351/60)	c(7361/70)	Per year

	**Dry Season Expenses**<br>[99] Don't know<br>[98] No response			**Wet Season Expenses**<br>[99] Don't know<br>[98] No response			
	**Min**	**Max**		**Min**	**Max**		CARD
Medications and other health related expenses (total)			Per month			Per month	(74)
Food			Per month			Per month	(75)
Livestock			Per month			Per month	(76)
Wages for hired labour (total)			Per month			Per month	(77)
Other (please specify) (e.g. rent, humanitarian aid etc)			Per year			Per month	(78)
	**c(11/20)**	**c(21/30)**		**c(31/40)**	**c(41/50)**		

65. If you went to the market to buy a unit of the following commodities, how much would you pay?

Charcoal:________ per basin / straw sacks *circle one*

Basin c(8011/17)

Straw c(8021/27)

Fuelwood__________________ per bundle

							c(8031/40)

66. Does your family rent oxen?
[1] Yes [0] No c(8108)

67. Does your family rent an ox plough?
[1] Yes [0] No c(8109)

68. Does your family hire somebody to plough your fields using oxen?
[1] Yes [0] No c(8110)

69. Does your family rent a tractor?
[1] Yes [0] No c(8111)

70. What are the 3 forest fruits that you collect most ofte

Fruit	Amount Dry Season<br>[1] A little bit<br>[2] More than a little<br>[3] A lot		Amount Wet season<br>[1] A little bit<br>[2] More than a little<br>[3] A lot	
(8112,14)	1 2 3	c(8131)	1 2 3	c(8141)
(8115,17)	1 2 3	c(8132)	1 2 3	c(8142)
(8118,20)	1 2 3	c(8133)	1 2 3	c(8143)
